# Short Peptides with Uncleavable Peptide Bond Mimetics as Photoactivatable Caspase-3 Inhibitors

**DOI:** 10.3390/molecules24010206

**Published:** 2019-01-08

**Authors:** Tim Van Kersavond, Raphael Konopatzki, Suravi Chakrabarty, Bernhard Blank-Landeshammer, Albert Sickmann, Steven H. L. Verhelst

**Affiliations:** 1 Leibniz-Institut für Analytische Wissenschaften ISAS, Otto-Hahn-Str. 6b, 44227 Dortmund, Germany; tim.kersavond@isas.de (T.V.K.); raphael.konopatzki@uni-duesseldorf.de (R.K.); bernhard.blank@isas.de (B.B.-L.); albert.sickmann@isas.de (A.S.); 2 Department of Cellular and Molecular Medicine, Laboratory of Chemical Biology, KU Leuven—University of Leuven, Herestraat 49 Box 802, 3000 Leuven, Belgium; suravi.chakrabarty@kuleuven.be

**Keywords:** chemical probes, click chemistry, inhibitors, photocrosslinkers, photoactivation, proteases, triazoles

## Abstract

Chemical probes that covalently interact with proteases have found increasing use for the study of protease function and localization. The design and synthesis of such probes is still a bottleneck, as the strategies to target different families are highly diverse. We set out to design and synthesize chemical probes based on protease substrate specificity with inclusion of an uncleavable peptide bond mimic and a photocrosslinker for covalent modification of the protease target. With caspase-3 as a model target protease, we designed reduced amide and triazolo peptides as substrate mimetics, whose sequences can be conveniently constructed by modified solid phase peptide synthesis. We found that these probes inhibited the caspase-3 activity, but did not form a covalent bond. It turned out that the reduced amide mimics, upon irradiation with a benzophenone as photosensitizer, are oxidized and form low concentrations of peptide aldehydes, which then act as inhibitors of caspase-3. This type of photoactivation may be utilized in future photopharmacology experiments to form protease inhibitors at a precise time and location.

## 1. Introduction

Proteases are enzymes that cleave proteins and peptides. They are not only involved in food digestion and protein turnover inside the cell, but also in highly regulated signaling cascades, such as blood coagulation [1] and regulated cell death (apoptosis) [2]. In order to prevent unchecked cleavage of substrates, protease activity is tightly regulated by various mechanisms [3], including zymogen activation, presence of endogenous inhibitors and post-translational modifications. The study of proteases has undergone invaluable progress by using chemical tools, such as protease inhibitors, synthetic substrates and covalent chemical probes [4,5].

**Figure 1:**
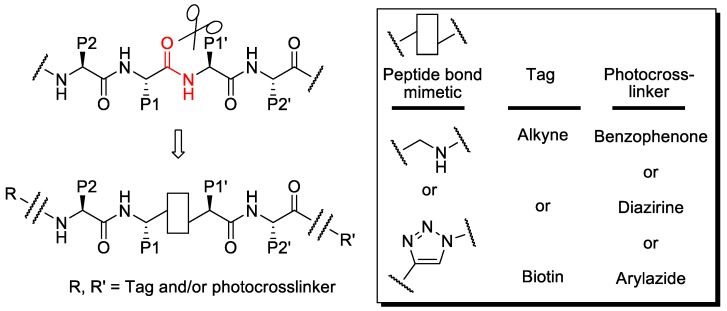
Molecular design of general protease-targeting photocrosslinking probes. A protease substrate (scissile bond in red, also indicated with scissors) is surrounded by amino acids in the non-primed site (N-terminally; P1, P2, etc.) and the primed site (P1′, P2′, etc.) that accommodate recognition by certain proteases. The scissile bond is replaced by a non-cleavable analogue, and a photocrosslinker and a tag are incorporated.

In the last two decades, the emergence of activity-based protein profiling (ABPP) has profoundly influenced the field of protease research [5,6,7,8]. ABPP makes use of different types of chemical probes that covalently label the active site of proteases. These include probes with various kinds of electrophiles that target catalytic residues of cysteine, serine and threonine proteases [9]. For proteases that utilize a nucleophilic water molecule for attack on the scissile peptide bond, other strategies have been put into place, such as the utilization of a combination of photocrosslinkers with metal-chelating peptide derivatives for labeling of metalloproteases [10,11,12]. In summary, the strategies to target different protease families with chemical probes are highly diversified. We wondered if a more general approach would be feasible. Obviously, a common feature of all proteases is affinity and catalytic activity towards their substrates. Most proteases display a certain degree of substrate specificity, which can be exploited for the generation of selective synthetic substrates either by using the natural substrate specificity or by incorporation of non-natural amino acids to increase selectivity amongst closely related protease family members [13]. We reasoned that a peptide with sufficient affinity towards the active site could potentially be used as a covalent probe, provided that (1) the scissile bond is replaced by a non-cleavable peptide bond mimic in order to prevent processing of the probe, and (2) a moiety is introduced for covalent modification, e.g., a photocrosslinker (Figure 1).

In this paper, we explore the above-mentioned strategy by using two different types of peptide bond mimetics (reduced amides, often abbreviated as ψ(CH_2_NH), and triazolo peptides) and various photocrosslinkers. Using caspase-3 as a target protease, we found that the reduced amide peptides can act as protease inhibitors upon irradiation. However, this did not lead to covalent modification of the target protease. Instead, it turned out that these peptides are activated upon irradiation leading to non-covalent protease inhibition. Hence, these molecules can potentially be utilized as photoactivatable inhibitors of caspases.

## 2. Results

For the construction of our probes, three different photocrosslinkers were selected: Benzophenone, diazirine and aryl azide, which are the most common classes of reagents for photoaffinity labeling, each with different reactivities and potential background labeling [14,15]. As peptide bond mimetics that are uncleavable by proteases, we chose reduced amides (ψ(CH_2_NH)) and triazoles for several reasons: (1) We envisioned that these would be synthetically accessible by modified Fmoc-based solid phase peptide synthesis (SPPS). (2) The building blocks necessary for the synthesis of these mimetics can be obtained in several steps from commercially available amino acid analogs. (3) The two different peptide bond mimetics have different properties: Whereas ψ(CH_2_NH) is more flexible than the scissile peptide bond, the triazole is more conformationally restricted and its strong dipole makes it a decent peptide bond mimic [16]. (4) The ψ(CH_2_NH) mimetic has been utilized before as protease inhibitor, for example for renin [17] and HIV protease [18].

### 2.1. Synthesis

**Scheme 1:**
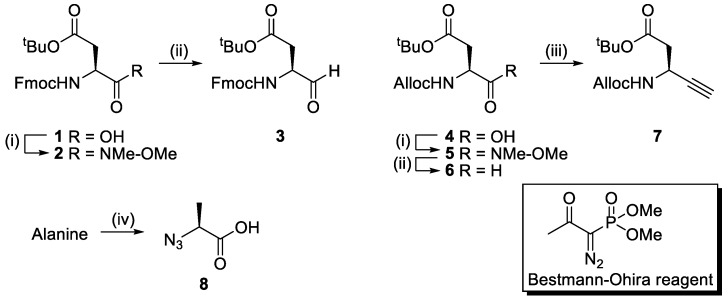
Construction of building blocks for solid phase peptide synthesis (SPPS) synthesis of ψ(CH_2_NH) and triazole uncleavable peptide bond mimetics. Reagents and conditions: (i) *N-*,*O*-dimethyl-hydroxylamine, HBTU, DIEA, dichloromethane (DCM). (ii) LiAlH_4_, THF. (iii) Bestmann–Ohira reagent, K_2_CO_3_, MeOH/MeCN. (iv) Imidazole-1-sulfonyl azide, K_2_CO_3_, CuSO_4_, MeOH.

In order to access peptides with a ψ(CH_2_NH) or a triazole as an uncleavable peptide bond mimetic, we aimed for a protocol compatible with Fmoc-based SPPS. We reasoned that construction of a ψ(CH_2_NH) would be possible by on-resin reductive amination, whereas triazole formation can be performed by on-resin click chemistry. To this end, aldehyde building block **3**, alkyne building block **7**, and azide building block **8** were required (see Scheme 1). Starting from commercially available Fmoc-Asp(tBu)-OH, Weinreb amide **2** was made by coupling to *N-*,*O*-dimethyl-hydroxylamine followed by lithiumaluminumhydride reduction to aldehyde **3** (Scheme 1). Seyfert–Gilbert homologation [19,20] of aldehyde **3** with the in situ generated Bestmann–Ohira reagent [21] turned out not to be compatible with the Fmoc protecting group. We therefore took a different protecting group strategy and utilized an Alloc group on the amino group of the aspartate (compound **4**), which was now uneventfully converted into alkyne **7**. The final building block, azide **8**, was made from l-alanine by a diazotransfer using imidazole-1-sulfonyl azide [22] (Scheme 1). 

With the different building blocks in hand, we set out to synthesize various probe molecules. The peptide sequence Asp-Glu-Val-Asp-Ala was chosen, because this is an optimal sequence for recognition by caspase-3, as shown in various studies [23,24,25,26]. These studies, which range from the synthesis of aminomethyl coumarin substrates [23] to mass spectrometry-based N-terminomics [24,25,26], found that the major determinant of specificity is the P1 Asp element and that other positions (P4–P2) contribute to the substrate specificity. Moreover, N-terminomics analysis identified small amino acid side chains in the P1′ to be most optimal.

**Scheme 2:**
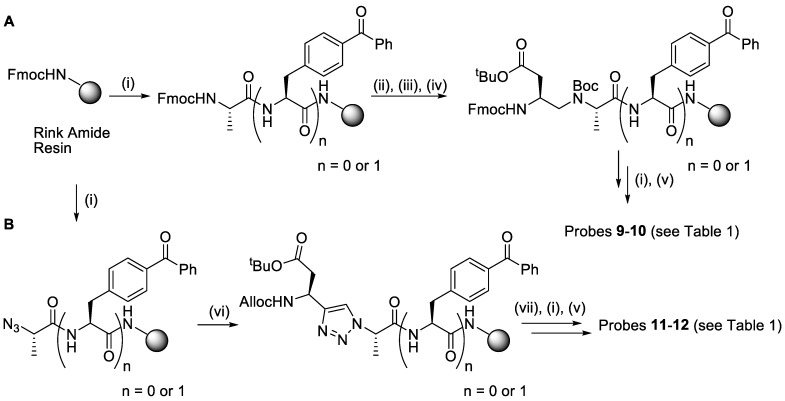
(**A**) Synthesis of ψ(CH_2_NH) probes containing a benzophenone photocrosslinker. (**B**) Synthesis of triazole peptide mimetics containing a benzophenone photocrosslinker Reagents and conditions: (i) Elongation: 20% piperidine in DMF, then coupling of amino acid with HBTU/DIEA. (ii) 20% piperidine in DMF, (iii) building block **3**, NaBH_3_CN, DMF/DCM/MeOH, AcOH, (iv) Boc_2_O, pyridine, DIEA. (v) TFA/H_2_O/TIS 95/2.5/2.5, (vi) CuBr, sodium ascorbate, 2,6-lutidine, DIEA, DMF/MeCN, (vii) PhSiH_3_, Pd(Ph_3_P)_4_, DCM.

For the ψ(CH_2_NH) peptides, Fmoc-deprotection of the P1′ alanine was followed by reductive amination with aldehyde **3** under the influence of sodium cyanoborohydride (Scheme 2). Successful reductive amination was confirmed by a negative Kaiser test and a positive chloranil test (see Materials and Methods for experimental details). Next, the secondary amine was protected with a Boc-group and the peptide sequence was elongated with standard Fmoc-based SPPS. Probes **9a**–**10c** were then cleaved from the resin and HPLC purified (Scheme 2 and Table 1).

For triazole-containing peptides, the P1′ alanine was coupled as azide building block **8**. On-resin Cu(I)-catalyzed azide-alkyne cycloaddition with building block **7** was achieved with copper(I) bromide in the presence of ascorbate, 2,6-lutidine and diisopropylethylamine (DIEA). This resulted in formation of the triazole moiety (Scheme 2). Completeness of the click chemistry reaction was confirmed by a modified Kaiser test, in which an aliquot of resin was first exposed to triphenyl phosphine and water to reduce potentially unreacted azides to amines [27]. Click reaction was followed by Alloc deprotection and standard Fmoc-based SPPS for elongation of the rest of the peptide sequence. Cleavage from the resin and HPLC purification yielded probes **11a** and **12a**. 

### 2.2. Inhibition Studies

**Figure 2:**
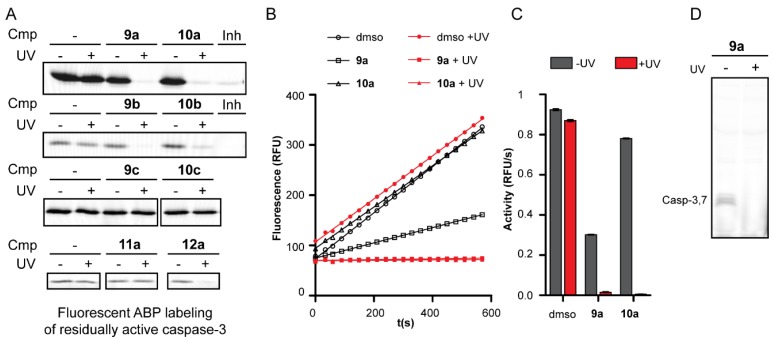
Inhibition of caspase-3. (**A**) Competitive activity-based protein profiling (ABPP) experiments using ψ(CH_2_NH) or triazolo peptide derivatives **9**–**12** with or without irradiation. Compounds **9a**–**12a** were used at 10 μM final concentration, **9b**, **9c**, **10b** and **10c** at 100 μM. Inh = caspase inhibitor biotin-DEVD-AOMK, used at 100 nM concentration. (**B**) Representative progress curves of fluorescence produced by Ac-DEVD-AMC (50 μM) cleavage by caspase-3, irradiated with or without 10 μM of the indicated compounds. (**C**) Bar graph of residual caspase-3 activity of replicate experiments (*n* = 2) of those in (**B**). (**D**) Inhibition of executioner’s caspase-3 and -7 in lysates of HEK293 cells, treated with cytochrome c/dATP to induce apoptosis.

To check whether our probes function as inhibitors, competitive ABPP [28] was performed. Recombinant caspase-3 was incubated with probes **9a**–**10c**, **11a** and **12a** with or without irradiation at 365 nm, followed by labeling of residually active caspase-3 with the fluorescent, caspase-directed activity-based probe (ABP) SV149, a tetramethylrhodamine derivative of KMB-01 [29]. Caspase activity itself was not affected by UV irradiation, as shown by bands of equal intensity with and without UV light (Figure 2A). For the ψ(CH_2_NH) peptides **9a** and **10a**, containing a benzophenone, as well as for **9b** and **10b**, containing a diazirine, irradiation led to complete inhibition of caspase activity, whereas the non-irradiated samples showed no decrease in caspase ABP labeling (Figure 2A; upper two panels). ψ(CH_2_NH) probes **9c** and **10c**, carrying a phenyl azide, however, did not show any inhibition upon irradiation, up to 100 μM concentration (Figure 2A). Whereas triazolo peptide **12a**, with the benzophenone in the P2′ position, displayed some inhibition upon irradiation at 10 μM concentration, compound **11a**, with the benzophenone in the P5 site, did not (Figure 2A). An increase of the triazole peptide probe concentration to 100 μM also did not lead to full inhibition (Supporting information, Figure S1) and therefore, attention in other experiments was focused on the ψ(CH_2_NH) derivatives. To confirm inhibition, we also performed substrate cleavage assays using the fluorogenic aminomethyl coumarin substrate Ac-DEVD-AMC. Here, we observed that compounds **9a** and **10a** display little to no inhibition, but that irradiation leads to complete blockage of substrate processing (Figure 2B,C). Inhibition of active caspases could also be achieved in an apoptotic cell lysate (Figure 2D).

### 2.3. Mechanism of Action

**Figure 3:**
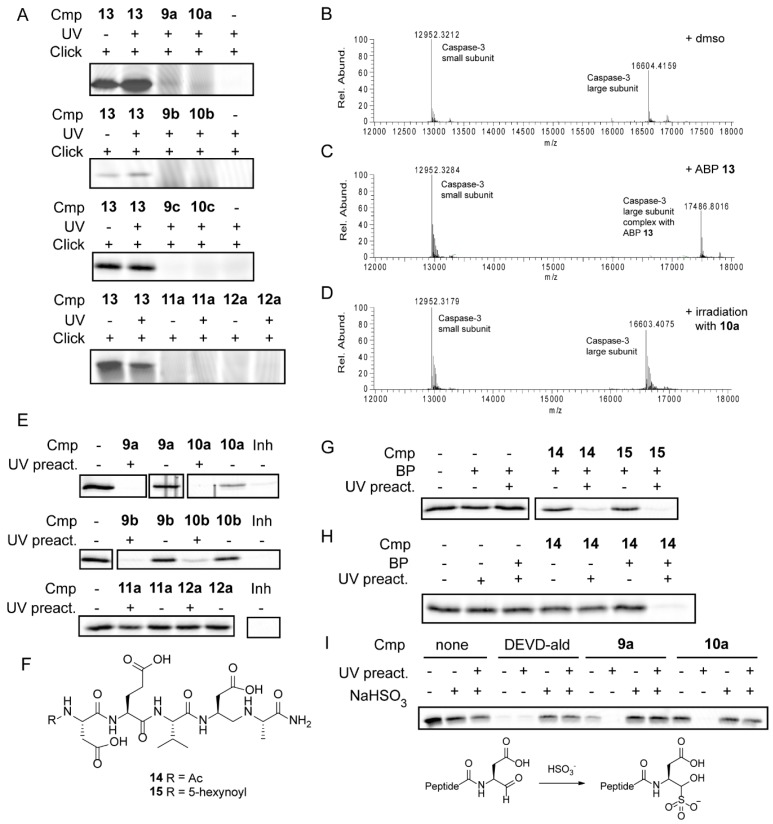
Non-covalent inhibition of caspase-3 by compounds **9** and **10**. (**A**) Fluorescent labeling of caspase-3 by ψ(CH_2_NH) peptides **9**–**10** and triazolo peptides **11**–**12**. Note that positive control probe **13** leads to strong, fluorescent labeling, but photocrosslinking peptides **9**–**12** do not. (**B**) Deconvoluted ESI-MS of caspase-3. (**C**) Deconvoluted ESI-MS of caspase-3 after incubation with covalent activity-based probe (ABP) **13**. Note that the active site, located on the large subunit, is modified. (**D**) Deconvoluted ESI-MS of caspase-3 after irradiation in the presence of benzophenone ψ(CH_2_NH) **10a**, not resulting in covalent modification. (**E**) Competitive ABPP shows inhibition of caspase labeling by “UV preactivation.” Note that inhibition is seen for ψ(CH_2_NH) peptides **9a**–**10b**, but not for triazole peptides **11**–**12**. (**F**) Structures of ψ(CH_2_NH) peptides **14** and **15** without photocrosslinker. (**G**) UV irradiation of ψ(CH_2_NH) peptides **14** and **15** in the presence of benzophenone leads to inhibition of caspase-3, as monitored by competitive ABPP. (**H**) Inhibition of caspase-3 by ψ(CH_2_NH) peptide **14** needs both BP and UV irradiation as shown by competitive ABPP. (**I**) Competitive ABPP shows trapping of active aldehyde species by creating a sodium bisulfite adduct using 10 mM NaHSO_3_. Ac-DEVD-aldehyde (100 nM) was used as a control compound, whereas **9a** and **10a** (10 μM) were irradiated for 10 min and reacted with sodium bisulfite for an additional 20 min before 30 min incubation with caspase-3 and read-out with a fluorescent caspase ABP.

Our probes were designed to irreversibly photocrosslink to caspase-3. In order to show covalent complex formation, we made use of the alkyne function located at the N-terminus of all probes by installing a tetramethyl rhodamine fluorophore (TAMRA) via copper(I)-catalyzed azide alkyne cycloaddition. Whereas a positive control probe (caspase-directed ABP **13** with an N-terminal alkyne tag; structure in Supporting Information, Figure S2) gave a clear, fluorescently labeled caspase-3 band, indicating a stable covalent bond formation, the ψ(CH_2_NH) and triazolo peptides **9**–**12** did not yield any covalent labeling (Figure 3A). To exclude that this observation was due to our gel-based read-out, we also performed mass spectrometry experiments. ESI-MS on purified caspase-3 showed the presence of the small and large subunit (Figure 3B). Treatment with covalent ABP **13** indicated a clear mass shift of the large subunit, on which the active site cysteine is located. This mass shift corresponded to the covalent modification (Figure 3C). However, incubation with benzophenone probe **10a** under irradiation at 365 nm did not reveal any modification (Figure 3D). MALDI-TOF MS (matrix-assisted laser desorption ionization time-of-flight mass spectrometry) experiments gave similar results for diazirine probe **9b** (Supporting information, Figures S3 and S4).

As these results indicate non-covalent inhibition, we performed a series of experiments to elucidate the mechanism of action. Compounds were irradiated with UV light of 365 nm for 30 min and after removal of the light source added to caspase-3. Residual caspase activity was measured by using a fluorescent caspase ABP as in our previous experiments above. To our surprise, caspase-3 was still inhibited in the case of the benzophenone probes **9a**–**10a** and diazirine probes **9b**–**10b** (Figure 3E), indicating the formation of a stable inhibitor instead of a short-lived biradical or carbene species. However, LC-MS analysis of these probes before and after irradiation did not show obvious changes, apart from the loss of nitrogen in the case of the diazirines **9b** and **10b** (Supporting Information, Figures S5–S8).

Interestingly, we found that the benzophenone moiety and the ψ(CH_2_NH) peptide mimic do not need to be present in the same molecule. When two ψ(CH_2_NH) peptides with the Asp-Glu-Val-Asp-ψ(CH_2_NH)-Ala motif (**14** and **15**, Figure 3F) were irradiated in the presence of an equimolar amount of benzophenone, this still led to inhibition (Figure 3G). The presence of a ψ(CH_2_NH) peptide with benzophenone or UV irradiation alone was not sufficient for inhibition, but all needed to be present to block the caspase-3 active site (Figure 3G,H). We hypothesized that the active species is a peptide aldehyde, formed by oxidation of the ψ(CH_2_NH) moiety (see also discussion in Section 3). To support this hypothesis, we performed an experiment in which this aldehyde was trapped by sodium bisulfite, forming a sodium bisulfite adduct. As shown in Figure 3I, Ac-DEVD peptide aldehyde efficiently inhibits caspase-3, but not when bisulfite is present. The irradiated samples with ψ(CH_2_NH) peptides **9a** and **10a** also lose their inhibitory capacity when exposed to sodium bisulfite (Figure 3I).

## 3. Discussion

The initial design of our molecules with ψ(CH_2_NH) or triazole uncleavable peptide bond mimetics was aimed at obtaining affinity-based probes that form a covalent bond to the target protease by means of a photocrosslinker. The peptide derivatives themselves do not compete with ABP binding or substrate cleavage, hence have very high IC_50_ values for caspase-3. This can be concluded from the competitive ABPP experiment that did not show any inhibition of caspase activity without irradiation (Figure 2A). As the read-out of this experiment utilizes a covalent caspase ABP, the reversible binding of the peptide derivatives may be ‘outcompeted’ over time by the covalent ABP. However, when the samples were irradiated, the ψ(CH_2_NH) peptides with benzophenone or diazirine photocrosslinkers (**9a**, **9b**, **10a** and **10b**) showed complete inhibition (Figure 2A). The arylazides **9c** and **10c** did not show any inhibition, which we attributed to reduction of the azide by dithiothreitol (DTT) in the buffer. Substrate turnover experiments (Figure 2B,C) confirmed low affinity of the ψ(CH_2_NH) peptides, which is in line with mid-micromolar K_M_ values of small molecule fluorogenic substrates. However, efficient inhibitory capacity was observed upon irradiation.

The triazolo peptides only showed inhibition when the benzophenone was located at the P2′ position (compound **12a**), but the activity was relatively weak. The triazole may cause a steric clash or a conformational restriction that is not well accepted by the active site of caspase-3 or with proteases altogether. This is supported by a recent study that used substrate-derived triazolo peptides as inhibitors for the cysteine cathepsins and found only very weak inhibition [30].

Although the ψ(CH_2_NH) peptides inhibited caspase-3 upon irradiation, installation of a fluorophore did not lead to fluorescent labeling of the targeted caspase-3 (Figure 3A). Contrary to our expectations, this pointed towards a non-covalent mechanism of inhibition, which was confirmed by mass spectrometry experiments on purified caspase-3 irradiated in absence or presence of the probes of interest (Figure 3B–D). 

In an attempt to elucidate the mechanism of inhibition, we performed a ‘preactivation’ experiment, in which ψ(CH_2_NH) or triazolo peptides were first irradiated with UV light, and only added to caspase-3 after removal of the light source. Surprisingly, this still led to inhibition for ψ(CH_2_NH) probes, but not for the triazole probes (Figure 3E). We concluded that the probe structure may have been modified upon UV irradiation, and we considered two options: (1) Irradiation leads to an internal reaction, creating a cyclic peptide, which displays high activity. (2) Irradiation leads to the formation of another, linear peptide species with high activity.

LC-MS analysis of ψ(CH_2_NH) probes with diazirine (**9b**–**10b**) before and after irradiation showed formation of a new product with a mass corresponding to the loss of nitrogen (Supporting Information, Figures S6 and S8). Although this points towards a cyclization rather than reaction with a water molecule, it is not clear whether it leads to a potent caspase inhibitor. For benzophenone ψ(CH_2_NH) probes **9a** and **10a**, irradiation did not show obvious changes (Supporting Information, Figures S5 and S7). Therefore, cyclization seemed improbable, as this would most likely lead to a shift in retention time. We hypothesized that irradiation could potentially lead to oxidation of a very small amount of the ψ(CH_2_NH) moiety with benzophenone as a photosensitizer, resulting in minute quantities of aldehydes that were not detected on LC-MS, but were sufficient for caspase-3 inhibition. Oxidation of secondary amines under influence of benzophenone has been reported before [31]. For ψ(CH_2_NH) probes, it will first lead to imine formation and eventually result in a peptide aldehyde, which is a highly potent electrophile directed against cysteine proteases. Note that triazoles cannot undergo this transformation, further explaining their inactivity. The oxidation hypothesis was supported by an experiment in which ψ(CH_2_NH) peptides **14** and **15**, not containing a benzophenone, were irradiated in the presence of 1 eq of spiked-in benzophenone (Figure 3G–I). Indeed, this led to inhibition of caspase-3, underlining that irradiation of the ψ(CH_2_NH) in presence of benzophenone is necessary, but that the two moieties do not need to be present within the same molecule.

**Scheme 3:**
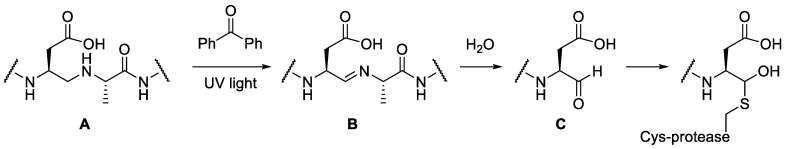
Proposed mechanism for photoinduced inhibition by ψ(CH_2_NH) compounds. Upon irradiation with UV light in the presence of benzophenone, a small amount of secondary amine (**A**) is oxidized to imine (**B**). Upon hydrolysis of this imine, peptide aldehyde (**C**) is formed, which is a potent inhibitor of cysteine proteases.

Sodium bisulfite reacts with aldehydes to form bisulfite adducts. Addition of this salt to the reaction mixture abolished the inhibition by **9a** and **10a** upon irradiation, further confirming the formation of the aldehyde as inhibitory species. We therefore propose a mechanism as depicted in Scheme 3: The excited benzophenone will—either through its biradical state or by formation of singlet oxygen—lead to oxidation of secondary amine A forming imine B. Hydrolysis of the imine will result in aldehyde C, which then acts as a strong electrophile, forming a reversible covalent bond with the active site cysteine of the targeted caspase-3.

Overall, this paper describes the unexpected finding of light-induced formation of cysteine protease inhibitors from substrate-based ψ(CH_2_NH) peptide derivatives. We provide evidence that this inhibition occurs through photo-induced oxidation leading to the formation of an aldehyde inhibitor. We imagine that this strategy may be used to induce the formation of cysteine protease inhibitors at a defined time and location for photopharmacological study of protease function.

## 4. Materials and Methods

### 4.1. Materials and Equipment

All materials were purchased from Sigma Aldrich GmbH (GmbH, Darmstadt, Germany), Carl Roth GmbH + Co. KG (Karlsruhe, Germany), Thermo Fischer Scientific Inc. (Dreieich, Germany), Rapp Polymere GmbH (Tübingen, Germany), Applichem GmbH (Darmstadt, Germany), VWR International GmbH (Darmstadt, Germany), Merck KGaA (Darmstadt, Germany), CreoSalus Inc. (Louisville, KY, USA), and Iris Biotech GmbH (Marktredwitz, Germany) and were used as received, unless otherwise noted. TLC was performed on pre-coated ALUGRAM SIL G plates with detection by a handheld UV lamp (254 nm) and subsequent staining with potassium permanganate, p-anisaldehyde, ninhydrin or cerium ammonium molybdate. LC-MS analysis was performed on a Thermo LCQ Fleet HPLC-MS/MS system using a gradient of 10% to 95% acetonitrile containing 0.1% formic acid. NMR spectra were recorded on a Bruker Avance DRX 400 spectrometer with tetramethylsilane as internal standard. High resolution ESI-FTMS was performed on a Thermo Scientific LTQ Orbitrap Velos Pro mass spectrometer.

### 4.2. General Procedures

#### 4.2.1. Kaiser Test

For the detection of residual primary amines on resin, a Kaiser test was performed after each peptide coupling step. Two drops of solution A (49 mL pyridine and 1 mL of a solution of 16.5 mg KCN in 25 mL H_2_O), solution B (5 g ninhydrin in 100 mL EtOH) and solution C (phenol:EtOH 4:1) were added to a glass tube. A small amount of resin was added, and the mixture was heated at 100 °C for 2 min. An almost colorless, clear solution indicated complete coupling. A dark blue solution indicates the presence of primary amines. For the detection of residual primary azides (after on resin click chemistry), an aliquot of resin was first treated with a 5% Ph_3_P solution in THF (*w*/*v*) for 15 min, washed with DMF, MeOH and dichloromethane (DCM), followed by a normal Kaiser test procedure.

#### 4.2.2. Chloranil Test

For the detection of residual secondary amines on resin, a chloranil test was performed when appropriate. Two drops of solution A (2% acetaldehyde in DMF) and solution B (2% chloranil in DMF) were added to a glass tube, to which a small amount of resin was added. After 5 min, a color change of the resin indicated the presence of primary (green) or secondary (blue) amines.

#### 4.2.3. General SPPS Elongation Procedure

Before loading of the first amino acid, polystyrene Rink amide resin was Fmoc-deprotected with 20% piperidine in DMF for 3 × 3 min. The resin was washed with DMF, MeOH and DCM. The first amino acid was coupled by using 3 eq. of Fmoc-protected amino acid, 3 eq. of HBTU and 6 eq. of DIEA in DMF. The mixture was shaken for 1 h after which the mixture was flushed away and the resin was washed with DMF, MeOH and DCM. Completion of the reaction was confirmed with a Kaiser test. For elongation, the same procedure for Fmoc-deprotection and amino acid coupling were performed.

#### 4.2.4. On Resin Reductive Amination

The resin-bound amino acid was Fmoc-deprotected with 20% piperidine in DMF for 3 × 3 min. Subsequently, the resin was washed with DMF, MeOH and DCM. Next, compound **3** (15 eq.; 1 M in DMF) was added to the resin. After 10 min, NaBH_3_CN (15 eq.; 1 M in DCM:MeOH 3:1 + 1% AcOH) was added. The reactor was shaken for 1 h, after which the resin was washed with DMF, MeOH and DCM. Completion of the reaction was confirmed with a Kaiser test (must be negative) and chloranil test (must be positive). The obtained resin was treated with di-*tert*-butyl dicarbonate (3 eq.; 0.4 M in DMF) and DIEA (3 eq.; 0.4 M in DMF). The reactor was shaken overnight, after which the resin was washed with DMF, MeOH and DCM. Completion of the reaction was confirmed with a Kaiser and chloranil test, which both must be negative.

#### 4.2.5. On Resin Click Chemistry

The resin was treated with DIEA (90 eq.), 2,6-lutidine (90 eq.), sodium ascorbate (36 eq., 0.5 M in DMF), CuBr (13 eq., 0.6 M in MeCN), and compound **7** (3 eq.). The reactor was shaken overnight, after which the mixture was flushed away and the resin washed with water, methanol, DMF, and DCM. A modified Kaiser test (see Section 4.2.1) was performed to confirm completion of the reaction.

#### 4.2.6. On Resin Alloc Deprotection

Phenylsilane (20 eq., 0.5 M) and tetrakis(triphenylphosphine)palladium (8 mol %) were dissolved in DCM. This mixture was added to the resin and the reactor was placed under Ar and shielded from light. After shaking for one hour, the mixture was drained, and this procedure was repeated twice. The resin was washed with DMF, methanol and DCM.

#### 4.2.7. Resin Cleavage and Purification

The resin was treated 3 × 30 min with a cleavage cocktail of TFA:TIS:water 95:2.5:2.5, and subsequently washed with a small amount of cleavage cocktail. The combined fractions were added to ice cold diethyl ether in order to precipitate the target compound. After centrifugation (5 min at 3000 *g*), most of the solvent was decanted. Any remaining volatiles were removed under a steady N_2_-stream. The crude product was purified with RP-HPLC using a linear gradient of MeCN in water with 0.1% TFA. Product containing fractions were combined and lyophilized.

### 4.3. Synthesis

*N-(9-fluorenylmethoxycarbonyl)-N’-methoxy-N’-methyl-l-isoasparagine t-butyl ester* (**2**). Fmoc-l-Asp(OtBu)-OH (3.0 mmol) and *N-*,*O*-dimethylhydroxylamine.HCl (4.5 mmol) were dissolved in DCM (15 mL) and cooled to 0 °C. DIEA (12 mmol), HBTU (4.5 mmol) and HOBt (4.5 mmol) were added to the reaction mixture. The mixture was placed under Ar, allowed to warm up to RT and stirred overnight. DCM was evaporated, and the residue was re-dissolved in 30 mL EtOAc and 30 mL water. The organic phase was washed with 5% HCl (30 mL), 1 M NaHCO_3_ (30 mL) and brine (30 mL). The organic layer was dried over MgSO_4_ and concentrated. The crude material was purified by column chromatography (petroleum ether:EtOAc 3:1) to give the title compound as a colorless oil (3.0 mmol, quantitative). ^1^H-NMR (500 MHz, Chloroform-d) δ 7.76 (d, *J* = 7.5 Hz, 2H), 7.60 (t, *J* = 8.4 Hz, 2H), 7.40 (t, *J* = 7.5 Hz, 2H), 7.30 (m, 2H), 5.73 (d, *J* = 9.1 Hz, 1H), 5.04 (t, *J* = 7.0 Hz, 1H), 4.36 (d, *J* = 7.3 Hz, 2H), 4.23 (t, *J* = 7.3 Hz, 1H), 3.80 (m, 3H), 3.24 (s, 3H), 2.74 (dd, *J* = 15.1, 5.4 Hz, 1H), 2.58 (dd, *J* = 15.1, 7.0 Hz, 1H), 1.45 (s, 9H). ^13^C-NMR (126 MHz, Chloroform-d) δ 170.9, 169.4, 155.6, 143.7, 141.2, 127.5, 126.9, 125.0, 119.8, 81.4, 67.0, 61.5, 48.3, 46.9, 38.1, 32.1, 27.8.

*N-(9-fluorenylmethoxycarbonyl)-l-aspartic aldehyde t-butyl ester* (**3**). Compound **2** (3.0 mmol) was dissolved in dry THF (30 mL), placed under Ar and cooled to 0 °C. LiAlH_4_ (3.3 mmol) was slowly added and the reaction mixture was stirred for 30 min at 0 °C. The reaction was quenched with saturated KHSO_4_ (30 mL). The THF was evaporated and the aqueous solution was extracted with EtOAc (150 mL). The organic phase was washed with brine (150 mL), dried over Na_2_SO_4_ and concentrated. The obtained crude **3** (2.30 mmol, 77%) was used without further purification. ^1^H-NMR (500 MHz, Chloroform-d) δ 9.65 (s, 1H), 7.77 (d, *J* = 7.6 Hz, 2H), 7.60 (d, *J* = 7.6 Hz, 2H), 7.41 (t, *J* = 7.5 Hz, 2H), 7.32 (t, *J* = 7.5 Hz, 2H), 5.89 (d, *J* = 8.3 Hz, 1H), 4.45 (m, 3H), 4.24 (t, *J* = 7.0 Hz, 1H), 2.97 (dd, *J* = 17.3, 4.7 Hz, 1H), 2.78 (dd, *J* = 17.3, 4.9 Hz, 1H), 1.45 (s, 9H). ^13^C-NMR (126 MHz, Chloroform-d) δ 198.9, 170.3, 156.1, 143.5, 141.4, 127.8, 127.1, 125.0, 120.1, 82.3, 67.3, 56.6, 47.1, 35.7, 28.0.

*N-(Allyloxycarbonyl)-l-aspartic acid β-t-butyl ester* (**4**). l-Asp(OtBu)-OH (5.3 mmol) and Alloc-OSu (7.9 mmol) were dissolved in THF (20 mL). K_2_CO_3_ (2.7 mmol, 10% in water) was added and the mixture was stirred overnight. After evaporation of THF, the mixture was diluted with 10% K_2_CO_3_ (10 mL) and washed four times with diethyl ether (30 mL). The aqueous phase was acidified with 5% HCl to pH 3 and extracted four times with DCM (30 mL). The combined organic layers were dried over MgSO_4_ and concentrated under reduced pressure. The obtained crude material was purified with column chromatography (DCM:methanol 200:1) to give the title compound (4.7 mmol, 89%). ^1^H-NMR (500 MHz, Chloroform-d) δ 6.65 (s, 1H), 5.90 (m, 1H), 5.77 (d, *J* = 8.6 Hz, 1H), 5.23 (dq, *J* = 10.5, 1.3 Hz, 2H), 4.60 (m, 3H), 2.99 (dd, *J* = 17.2, 4.4 Hz, 1H), 2.77 (m, 1H), 1.44 (s, 9H). ^13^C-NMR (126 MHz, Chloroform-d) δ 175.5, 170.3, 156.0, 132.4, 118.0, 82.4, 66.1, 53.5, 37.6, 28.0.

*N-(Allyloxycarbonyl)-l-aspartic aldehyde t-butyl ester* (**6**). Compound **4** (4.7 mmol) and *N*,*O*-dimethylhydroxylamine.HCl (7.1 mmol) were dissolved in DCM (20 mL) and cooled to 0 °C. DIEA (14.1 mmol), HBTU (7.1 mmol) and HOBt (7.1 mmol) were added to the reaction mixture. The mixture was placed under Ar, allowed to warm up to RT and stirred overnight. The DCM was evaporated, and the residue was re-dissolved in 30 mL EtOAc and 30 mL water. The organic phase was washed with 5% HCl (30 mL), 1 M NaHCO_3_ (30 mL) and brine (30 mL). The organic layer was dried over MgSO_4_ and concentrated. The crude material was purified by column chromatography (petroleum ether:EtOAc 3:2) to give the Weinreb amide as a colorless oil (3.3 mmol, 70%). The Weinreb amide (1.7 mmol) was dissolved in dry THF (20 mL), placed under Ar and cooled to 0 °C. LiAlH_4_ (2.0 mmol) was slowly added and the reaction mixture was stirred for 30 min at 0 °C. The reaction was quenched with saturated KHSO_4_ (20 mL). The THF was evaporated and the aqueous solution was extracted with EtOAc (100 mL). The organic phase was washed with brine (100 mL), dried over Na_2_SO_4_ and concentrated. The crude title compound (0.9 mmol, 53%) was used without further purification.

*(3S)-3-N-(Allyloxycarbonyl)amino-pent-4-ynoic acid t-butyl ester* (**7**). Dimethyl 2-oxopropylphosphonate (1.1 mmol) and K_2_CO_3_ (4.0 mmol) were dissolved in MeCN (9 mL). Imidazole-1-sulfonyl-azide. HCl (1.2 mmol), prepared according to a literature procedure [22], was added to the mixture, which was placed under Ar and stirred for 2 h. Compound **6** (0.9 mmol) was dissolved in methanol (9 mL) and added to the brown-yellow mixture and it was stirred overnight. The mixture was filtered, and the filter cake was washed three times with ethyl acetate. The clear filtrate was concentrated and resuspended in water (10 mL). The aqueous suspension was extracted three times with EtOAc (10 mL). The combined organic phases were washed with brine (20 mL), dried over MgSO_4_ and concentrated under reduced pressure. The crude material was purified by column chromatography (petroleum ether:EtOAc 3:1) to give the title compound (0.16 mmol, 18%). ^1^H-NMR (500 MHz, Chloroform-d) δ 5.91 (ddq, *J* = 16.7, 11.4, 5.7 Hz, 1H), 5.65 (d, *J* = 9.5 Hz, 1H), 5.23 (ddq, *J* = 10.5, 2.9, 1.4 Hz, 2H), 4.82 (dd, *J* = 26.6, 7.3 Hz, 1H), 4.61–4.54 (m, 2H), 2.84–2.57 (m, 2H), 2.31 (dd, *J* = 7.1, 2.4 Hz, 1H), 1.50–1.36 (m, 9H). ^13^C-NMR (126 MHz, Chloroform-d) δ 169.5, 155.2, 132.6, 118.0, 81.8, 81.7, 71.4, 65.9, 40.9, 39.8, 28.1.

*Azido-l-alanine* (**8**). l-Ala-OH (0.8 mmol), CuSO_4_ (0.008 mmol, cat.), and K_2_CO_3_ (2.2 mmol) were dissolved in methanol (40 mL) and placed under Ar. Imidazole-1-sulfonyl-azide.HCl (1.0 mmol) was added and the mixture was stirred overnight. The mixture was concentrated, and the residue was re-dissolved in 5% HCl (20 mL). The aqueous phase was extracted three times with ethyl acetate (20 mL). The organic phase was dried over MgSO_4_ and concentrated. The crude material was purified by column chromatography (petroleum ether:ethyl acetate 10:1 + 1% acetic acid) to give the title compound (0.37 mmol, 47%) as a yellow oil. ^1^H-NMR (500 MHz, DMSO-d_6_) δ 9.78 (d, *J* = 7.3 Hz, 1H), 4.31 (q, *J* = 7.4 Hz, 1H), 1.36 (d, *J* = 7.4 Hz, 3H). ^13^C-NMR (126 MHz, DMSO-d_6_) δ 172.9, 48.6, 16.6.

*Hexynoyl-Bpa-Asp-Glu-Val-Aspψ(CH_2_NH)-Ala-NH_2_* (**9a**). The title compound was synthesized according to the general procedure and isolated after HPLC purification as a white solid (0.80 μmol, 4% overall yield). HRMS (ESI) calcd. 878.3931 [M + H]^+^, found 878.3918.

*Hexynoyl-photoLeu-Asp-Glu-Val-Aspψ(CH_2_NH)-Ala-NH_2_* (**9b**). The title compound was synthesized according to the general procedure and isolated after HPLC purification as a white solid (0.80 μmol, 7% overall yield). HRMS (ESI) calcd. 752.3574 [M + H]^+^, found 752.3572.

*Hexynoyl-p-azido-Phe-Asp-Glu-Val-Aspψ(CH_2_NH)-Ala-NH_2_* (**9c**). The title compound was synthesized according to the general procedure and isolated after HPLC purification as a white solid (0.12 μmol, 1% overall yield). HRMS (ESI) calcd. 815.3683 [M + H]^+^, found 815.3678.

*Hexynoyl-Asp-Glu-Val-Aspψ(CH_2_NH)-Ala-Bpa-NH_2_* (**10a**). The title compound was synthesized according to the general procedure and isolated after HPLC purification as a white solid (0.80 μmol, 7% overall yield). HRMS (ESI) calcd. 878.3931 [M + H]^+^, found 878.3916.

*Hexynoyl-Asp-Glu-Val-Aspψ(CH_2_NH)-Ala-photoLeu-NH_2_* (**10b**). The title compound was synthesized according to the general procedure and isolated after HPLC purification as a white solid (0.93 μmol, 8% overall yield). HRMS (ESI) calcd. 752.3574 [M + H]^+^, found 752.3560.

*Hexynoyl-Asp-Glu-Val-Aspψ(CH_2_NH)-Ala-p-azido-Phe-NH_2_* (**10c**). The title compound was synthesized according to the general procedure and isolated after HPLC purification as a white solid (1.23 μmol, 11% overall yield). HRMS (ESI) calcd. 815.3683 [M + H]^+^, found 815.3673.

*Hexynoyl-Bpa-Asp-Glu-Val-Asp(triazolo)-Ala-NH_2_* (**11a**). The title compound was synthesized according to the general procedure and isolated after HPLC purification as a white solid (0.33 μmol, 2% overall yield). HRMS (ESI) calcd. 916.3836 [M + H]^+^, found 916.3830.

*Hexynoyl-Asp-Glu-Val-Asp(triazolo)-Ala-Bpa-Gly-NH_2_* (**12a**). The title compound was synthesized according to the general procedure and isolated after HPLC purification as a white solid (2.57 μmol, 13% overall yield). HRMS (ESI) calcd. 973.4050 [M + H]^+^, found 973.4046.

*Acetyl-Asp-Glu-Val-Aspψ(CH_2_NH)-Ala-NH_2_* (**14**). The title compound was synthesized according to the general procedure and isolated after HPLC purification as a white solid (4.97 μmol, 27% overall yield). HRMS (ESI) calcd. 575.2672 [M + H]^+^, found 575.2664.

*Hexynoyl-Asp-Glu-Val-Aspψ(CH_2_NH)-Ala-NH_2_* (**15**). The title compound was synthesized according to the general procedure and isolated after HPLC purification as a white solid (4.06 μmol, 22% overall yield). HRMS (ESI) calcd. 627.2985 [M + H]^+^, found 627.2975.

### 4.4. Biochemistry

#### 4.4.1. Gel-Based Competitive ABPP

Caspase-3, produced in *E. coli* and purified as described before [32], was reacted in PBS with 1 mM DTT. Samples were combined with the indicated probe and irradiated with a handheld UV lamp at 365 nm for 30 min at room temperature. For competition experiments with ‘pre-activation,’ the indicated probes were irradiated for 30 min prior to addition to caspase-3, and incubated for an additional 30 min. Next, ABP **13** was added (final concentration 1 μM) to label residual caspase-3 activity. The samples were incubated for 30 min at room temperature, after which 1/3rd volume of 4× Laemmli buffer was added. Samples were heated at 95 °C for 2 min and resolved by 15% SDS-PAGE. Gels were scanned using a Typhoon Trio+ fluorescent scanner with excitation at 532 nm and an emission filter of 580 nm. Gel images were processed with ImageJ (background subtraction with rolling ball radius of 50 pixels and automated contrast adjustment).

#### 4.4.2. Protease Kinetics Experiments

Purified caspase-3 (2 nM) was pre-activated by incubating in caspase reaction buffer (20 mm HEPES pH 7.4, 100 mM NaCl, 1 mM EDTA, 10% sucrose (*w*/*v*), 0.1% CHAPS (*w*/*v*), 10 mM DTT) for 15 min at 37 °C. Inhibitors **9a**, **10a**, and DMSO (control) were incubated with the caspase-3 at 10 μM final concentration. One set was irradiated with a handheld UV lamp at 365 nm for 30 min at room temperature whereas the control set was not irradiated. After 30 min, 99 μL of the sample was added to 1 μL of Ac-DEVD-AMC fluorogenic substrate (5 mM in dmso; 50 μM final concentration) in a black 96 well plate. The fluorescence intensity was read using an iD3 SpectraMax platereader (Molecular Devices; excitation wavelength: 340 nm; emission wavelength: 475 nm). The experiment was performed in duplicate. Activity was determined as the slope from the linear part of the progress curve by using GraphPad Prism.

#### 4.4.3. Fluorescent Labeling Experiments

Probes were clicked onto 5-TAMRA-azide (Carl Roth, Germany) by using the following conditions: To PBS, the probe (at twice the indicated concentration) was added, together with 5-TAMRA-azide (25 μM), Tris(3-hydroxypropyltriazolylmethyl)amine (THPTA, 50 μM), CuSO_4_ (1 mM), and sodium ascorbate (1 mM). After 60 min incubation at room temperature, this was then added to an equal volume of purified caspase-3 in PBS with 2 mM DTT, and irradiated with a handheld UV lamp at 365 nm for 30 min. 1/3rd volume of 4× Laemmli buffer was added, and the samples were processed for gel analysis as described above.

#### 4.4.4. ESI-MS on Caspase-3

Intact proteins were analyzed on an orbitrap Fusion Lumos mass spectrometer equipped with a nanoelectrospray ion source (Thermo Fischer, Dreieich, Germany). Sample desalting was performed using Amicon Ultra-0.5 3K NMWL centrifugal filters (Merck Millipore, Darmstadt, Germany) according to the manufacturer’s protocol and samples were brought to a final concentration of approx. 1 pmol/µL (in 25% MeOH and 0.5% formic acid). Analysis was performed by directly infusing the samples using a 25 µL syringe (Hamilton, Reno, NV, USA) operated by a syringe drive maintaining a flow rate of 300 nL/min. The mass spectrometer was operated in intact protein mode with the nitrogen pressure set to 3 mtorr in the ion-routing multipole. S-Lens RF-level was set to 80% and in source fragmentation was kept at 0 V. Spectra were acquired in the orbitrap with 10 microscans at a resolving power of 240,000 (at 200 *m*/*z*) with the automatic gain control (AGC) target set to 1 × 10^6^. Spectra were deconvoluted using the Xtract algorithm of the Xcalibur suite (Thermo Scientific). Resolving power was set to 180,000 (at 400 *m*/*z*), minimum signal to noise (S/N) threshold was 4 and the maximum allowed charge state 20. 

#### 4.4.5. MALDI-MS on Caspase-3

A total of 10 mM DTT was added to a stock solution of caspase-3 (200 ng/μL; 100 mM TRIS, pH 8.0, 100 mM NaCl), and this was treated with 10 vol % of the indicated probe (1 mM) and irradiated with a handheld UV lamp at 365 nm for 30 min. Afterwards, the samples were diluted in 0.1% TFA in water (20 ng/μL final concentration of caspase-3). Then, 1 μL of sinapic acid solution A (saturated sinapic acid in ethanol) was spotted on a BRUKER MTP 385 ground steel TF target plate, followed by 1 μL of a mixture of equal volumes of the caspase-3 sample and sinapic acid solution B (saturated sinapic acid in 3:7 acetonitrile:0.1% TFA in water). The MALDI spectra were recorded with a BRUKER ultrafleXtreme MALDI-TOF/TOF system.

#### 4.4.6. Competitive ABPP with Bisulfite Aldehyde Quenching

Compounds were incubated with or without irradiation (10 min) in PBS with or without 10 mM NaHSO_3_ and incubated for another 20 min before addition to caspase-3. After 30 min, ABP **13** was added (final concentration 1 μM) to label residual caspase activity. The samples were incubated for 30 min at room temperature, after which 1/3rd volume of 4× Laemmli buffer was added. Samples were processed in the same way as under Section 4.4.1.

## Figures and Tables

**Table 1:** Overview of synthesized ψ(CH_2_NH) and triazole peptides.

Compound	Structure ^1^	Type
**9a**–**c**	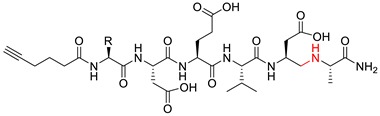	ψ(CH_2_NH)
**10a**–**c**	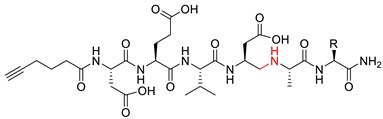	ψ(CH_2_NH)
**11a**	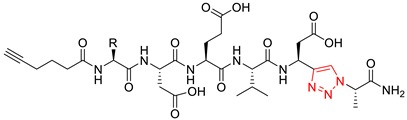	triazolo
**12a**	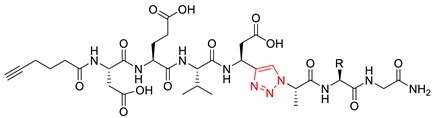	triazolo
	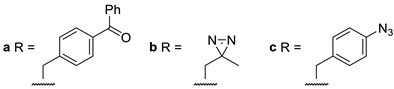	

^1^ Uncleavable peptide bond mimic, replacing the scissile bond, is represented in red.

## Supplementary Materials

The following are available online: Figures S1–S8.
