# Supplementary material for: Short Peptides with Uncleavable Peptide Bond Mimetics as Photoactivatable Caspase-3 Inhibitors
Source: Molecules. 2019 Jan 8;24(1):206. doi: 10.3390/molecules24010206 (PMC6337261; doi:10.3390/molecules24010206)
Supplement: Supplementary file 1 [file molecules-24-00206-s001.pdf]

Supporting Information

Short peptides with uncleavable peptide bond mimetics as photoactivatable caspase-3 inhibitors

Tim Van Kersavond [1], Raphael Konopatzki [1], Suravi Chakrabarty [2], Bernard Blank-Landeshammer [1], Albert Sickmann [1] and Steven H. L. Verhelst [1,2,*]
[1] Leibniz-Institute for Analytical Sciences ISAS, AG Chemical Proteomics, Otto-Hahn-Str. 6b, 44227 Dortmund, Germany; steven.verhelst@isas.de
[2] KU Leuven – University of Leuven, Laboratory of Chemical Biology, Department of Cellular and Molecular Medicine, Herestraat 49 box 802, 3000 Leuven, Belgium; steven.verhelst@kuleuven.be

Content Page

Figure S1 – inhibition of caspase-3 with triazolo peptides at 100 μM S2
Figure S2 – structure of alkyne ABP **13** S3
Figure S3 – MALDI-TOF spectrum of caspase-3 + UV irradiation S4
Figure S4 – MALDI-TOF spectrum of caspase-3 and compound **9b** + UV irradiation S5
Figure S5 – LC-MS data of compound **9a**, before and after UV irradiation S6
Figure S6 – LC-MS data of compound **9b**, before and after UV irradiation S7
Figure S7 – LC-MS data of compound **10a**, before and after UV irradiation S8
Figure S8 – LC-MS data of compound **10b**, before and after UV irradiation S9
Copies of NMR spectra S10
Copies of LC-MS data S15

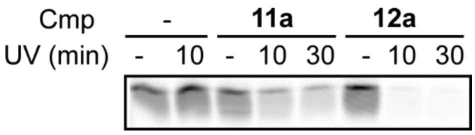

Figure S1. Competitive ABPP on caspase-3 using triazolo peptides **11a** and **12a** at 100 µM. Although compound **12a** displays inhibition that is nearly quantitative, compound **11a** does not show efficient inhibition.

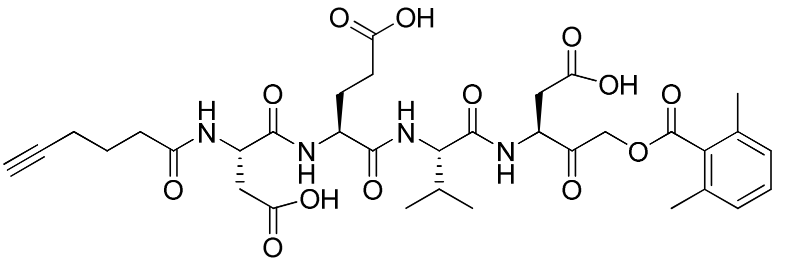

13

Figure S2. Caspase-directed ABP **13** with a clickable alkyne handle at the N-terminus.

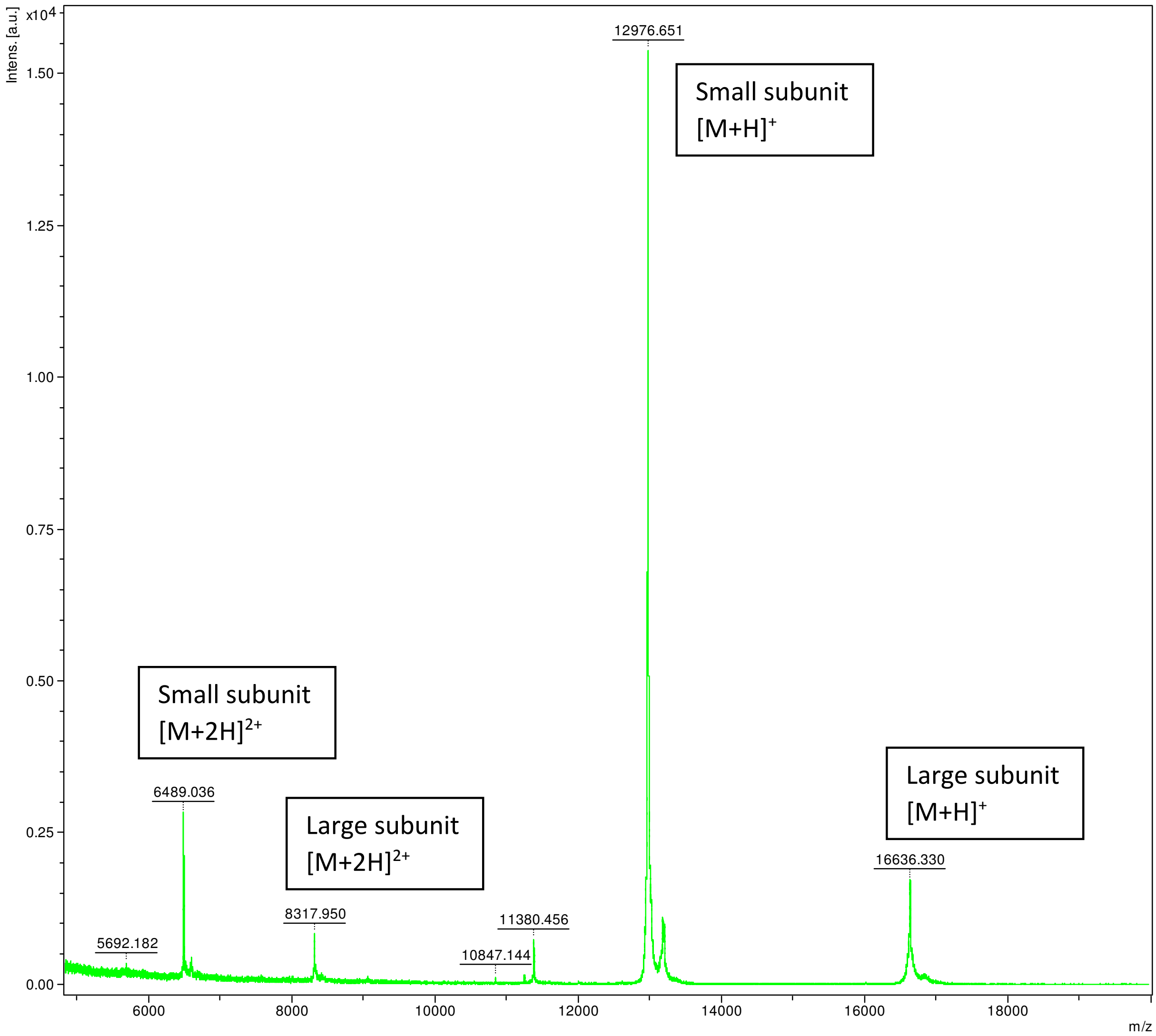

Figure S3. MALDI-TOF spectrum of purified, recombinant caspase-3 after 30 min of UV irradiation. Both the small and large subunit were detected.

Small subunit:

```
SGVDDDMACH KIPVEADFLY AYSTAPGYYS WRNSKDGSWF IQSLCAMLKQ YADKLEFMHI
LTRVNRKVAT EFESFSFDAT FHAKKQIPCI VSMLTKELYF YHLEHHHHHH
```

Monoisotopic mass: 12952.23; Average mass: 12960.73. Indicated mass likely corresponds to one methionine oxidation.

Large subunit (propeptide in grey):

```
MENTENSVDS KSIKNLEPKI IHGSESMDSG ISLDNSYKMD YPEMGLCIII NNKNFHKSTG
MTSRSGTDVD AANLRETFRN LKYEVRNKND LTREEIVELM RDVSKEDHSK RSSFVCVLLS
HGEEGIIFGT NGPVDLKKIT NFFRGDRCRS LTGKPKLFII QACRGTELDC GIETD
```

Monoisotopic mass: 16604.27; Average mass: 16614.85. Indicated mass likely corresponds to one methionine oxidation.

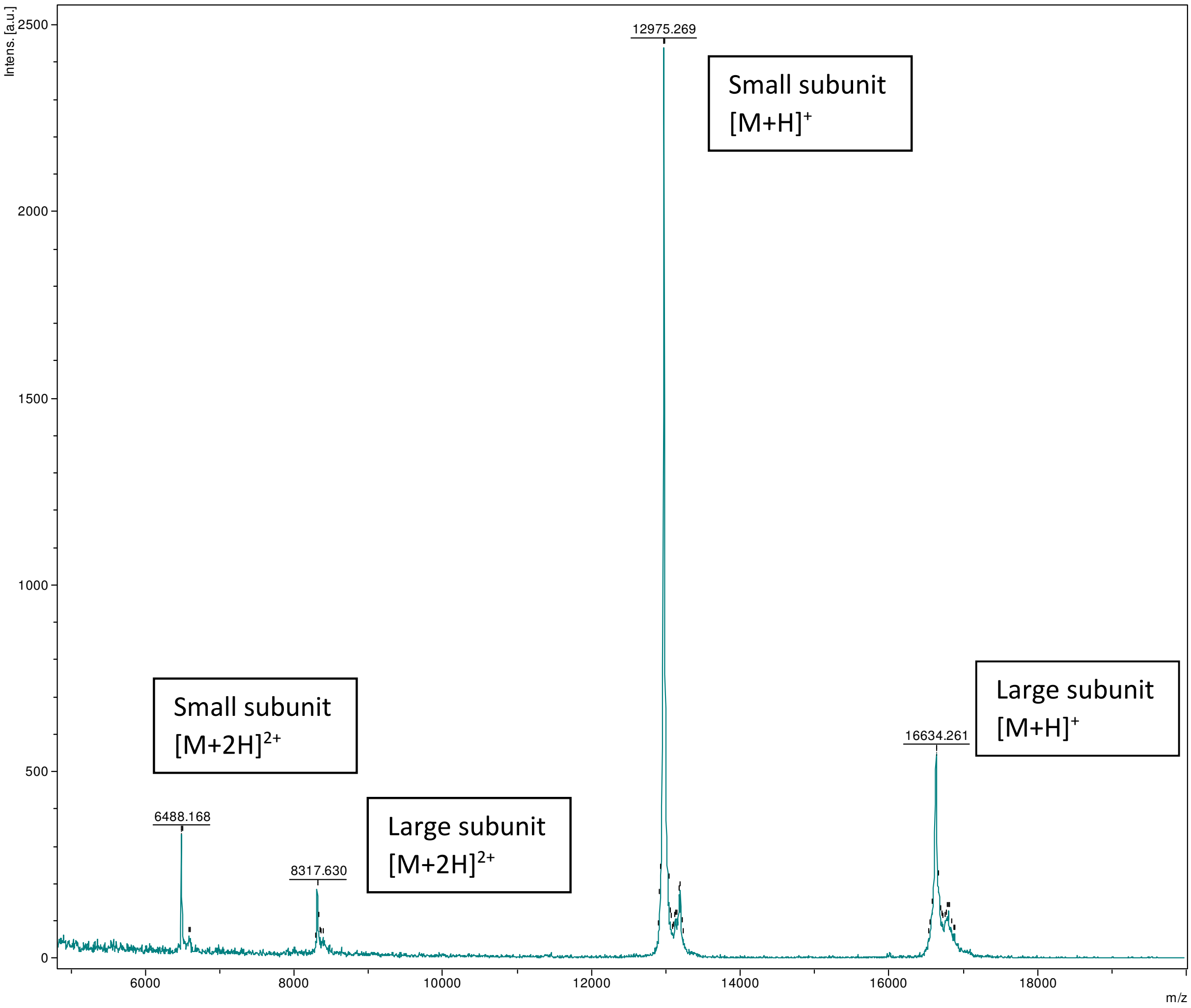

Figure S4. MALDI-TOF spectrum of purified, recombinant caspase-3 after 30 min of UV irradiation in the presence of diazirine probe **9b**. No shifts in the mass of the subunits is detected compared with the caspase-3 alone (compare with Figure S3).

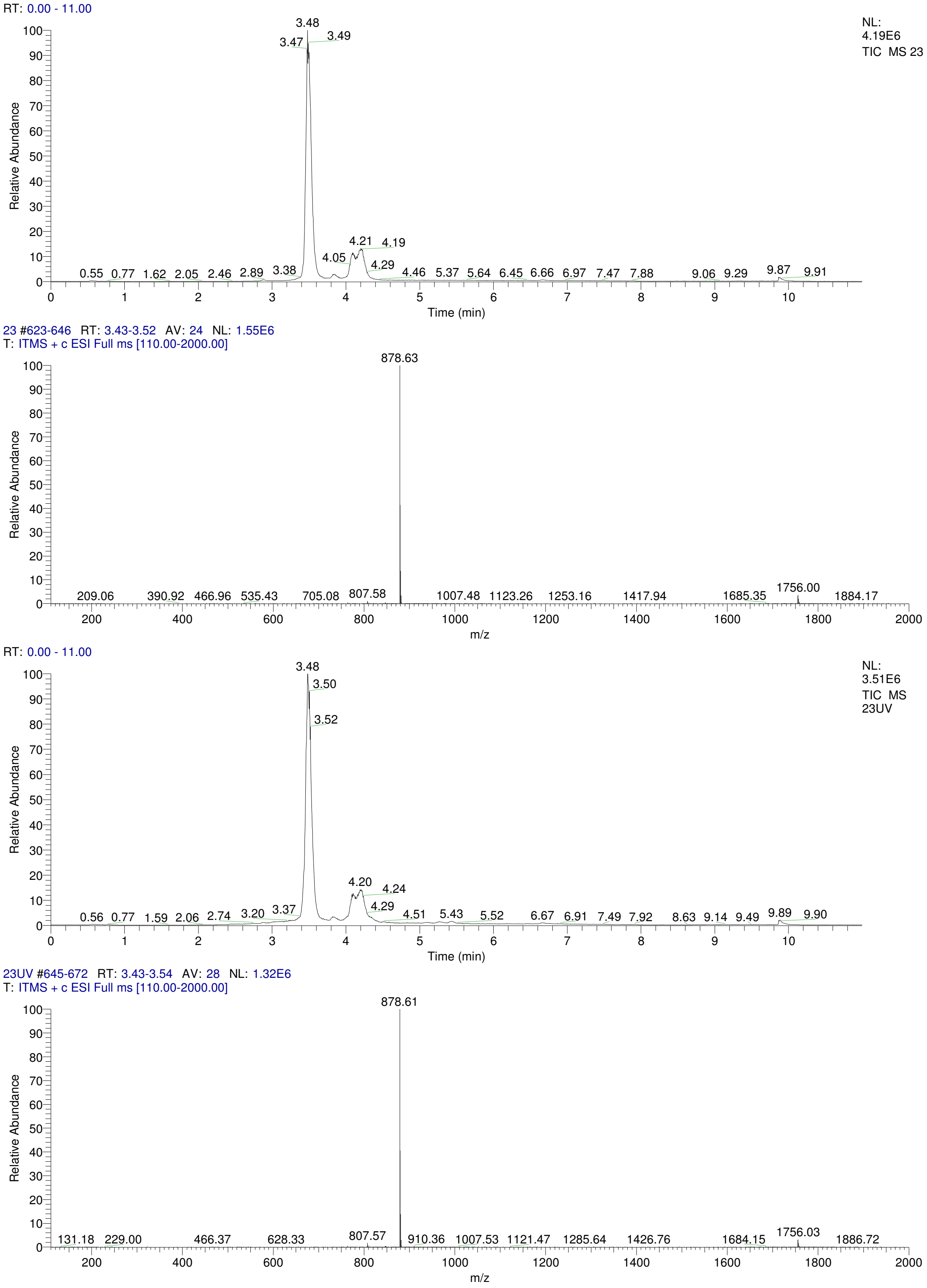

Figure S5. LC-MS data of compound **9a**, before and after UV irradiation.

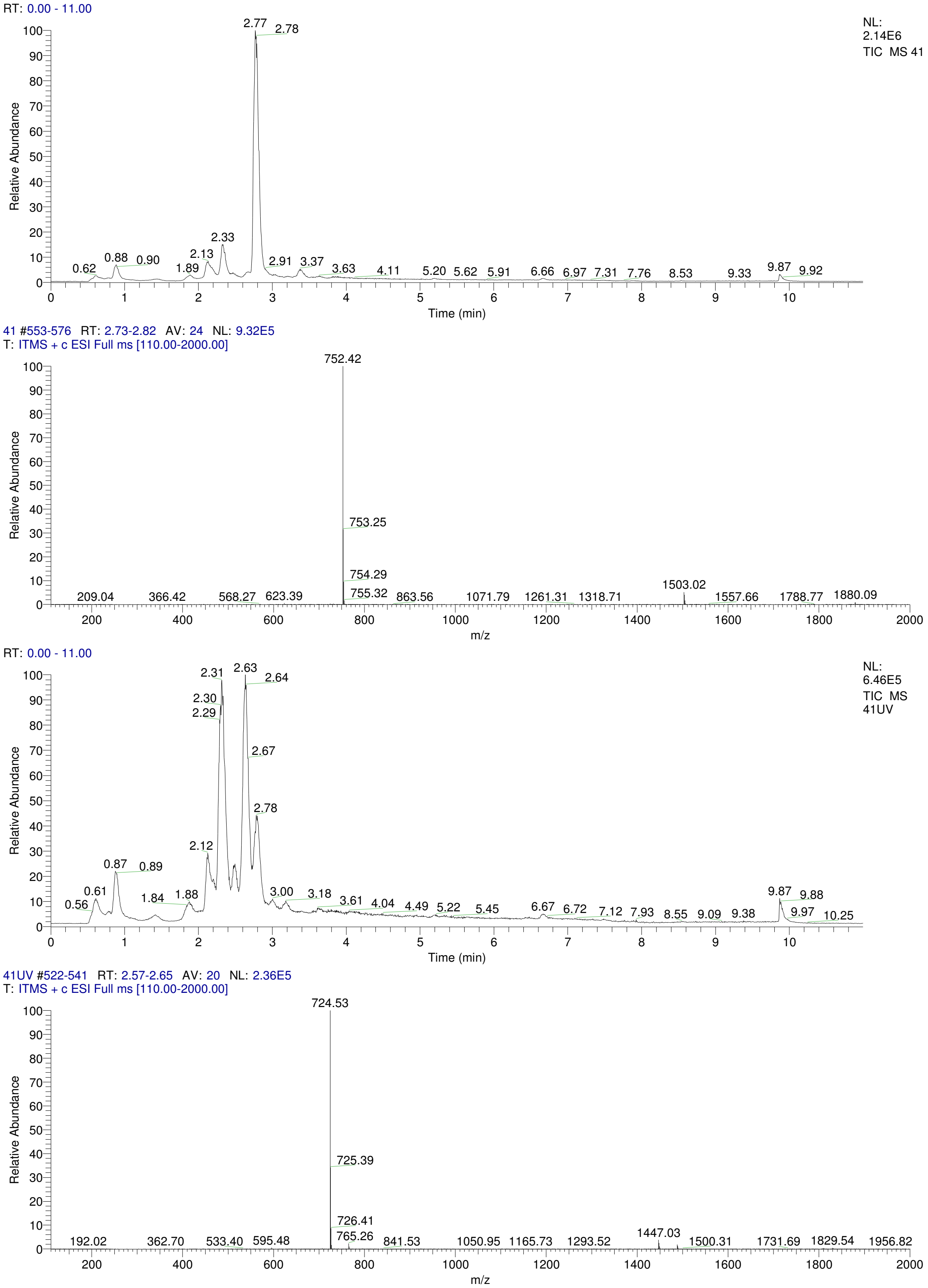

Figure S6. LC-MS data of compound **9b**, before and after UV irradiation

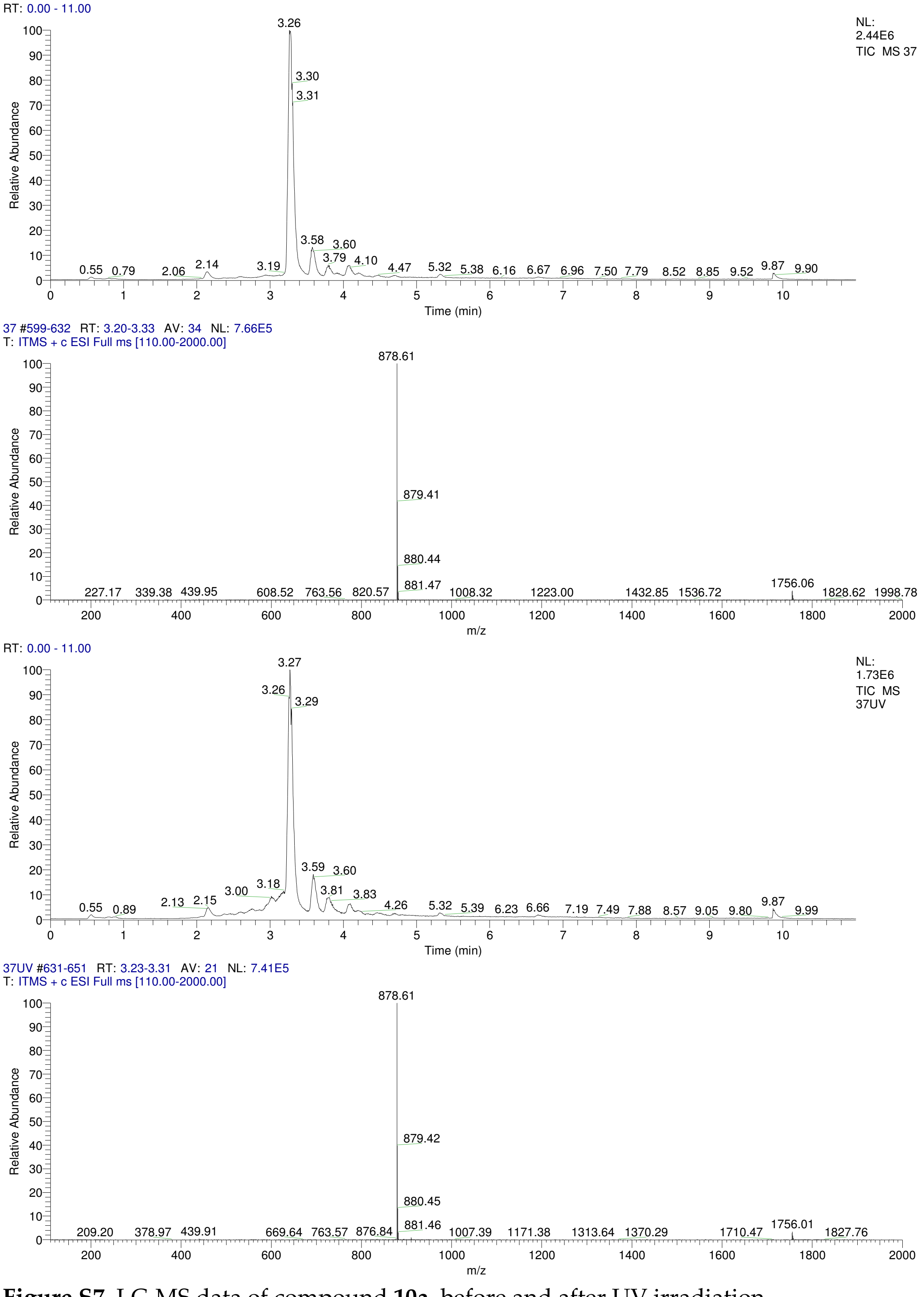

Figure S7. LC-MS data of compound **10a**, before and after UV irradiation

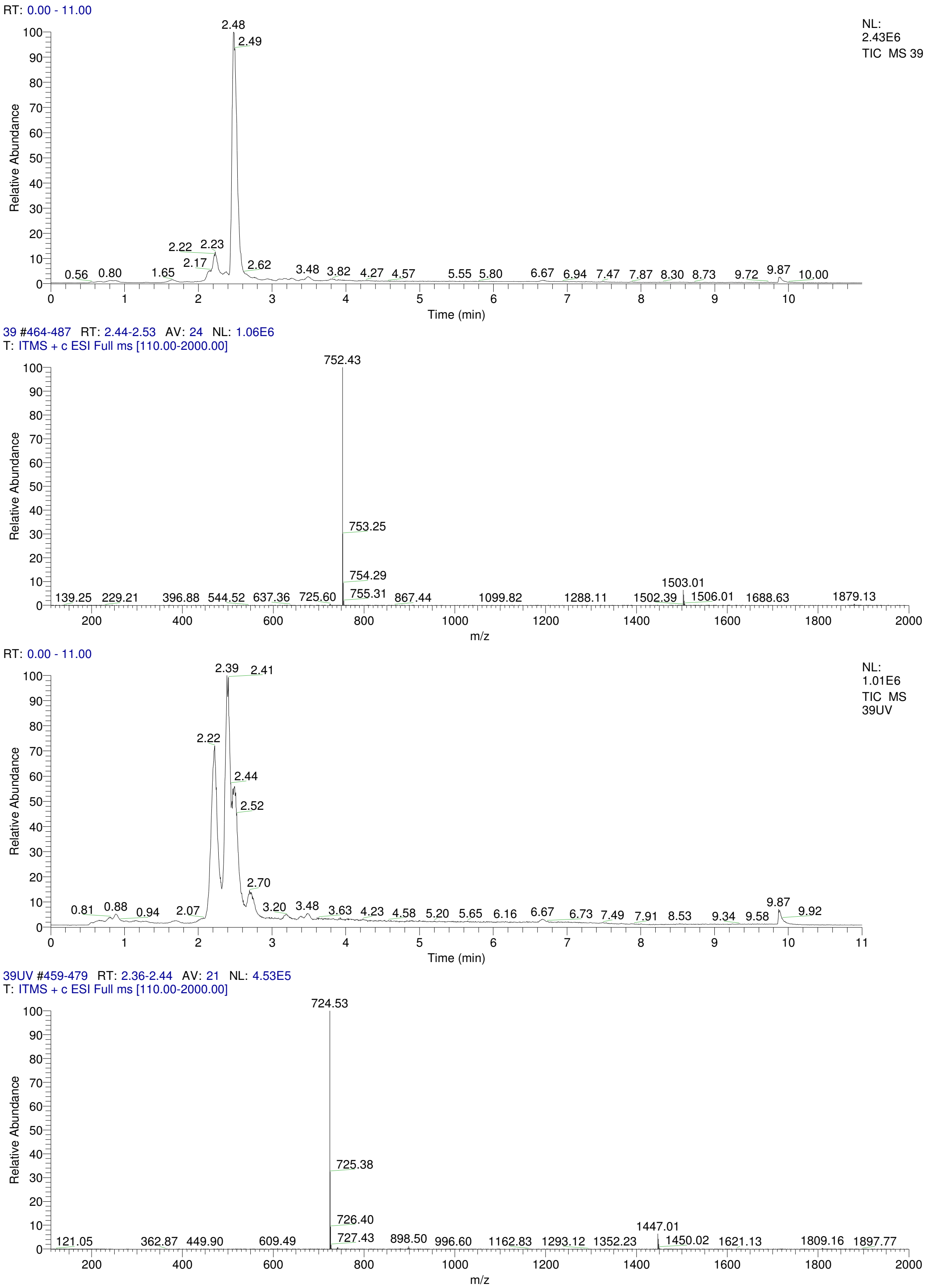

Figure S8. LC-MS data of compound **10b**, before and after UV irradiation

Copies of NMR spectra

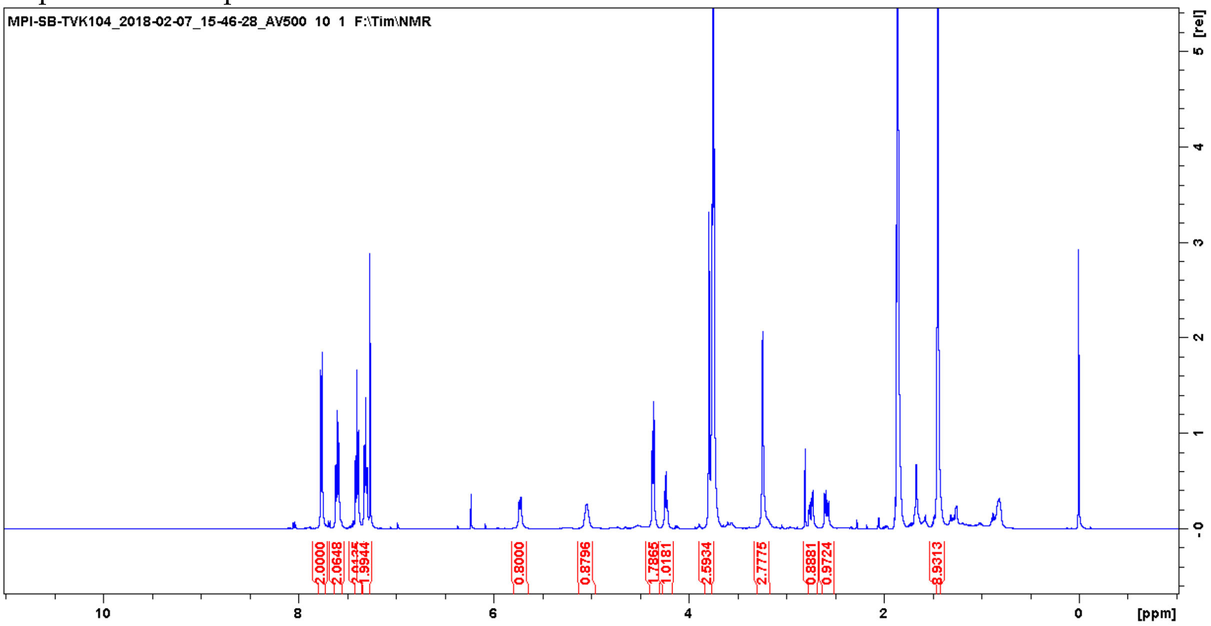

^1H NMR spectrum of compound **2**

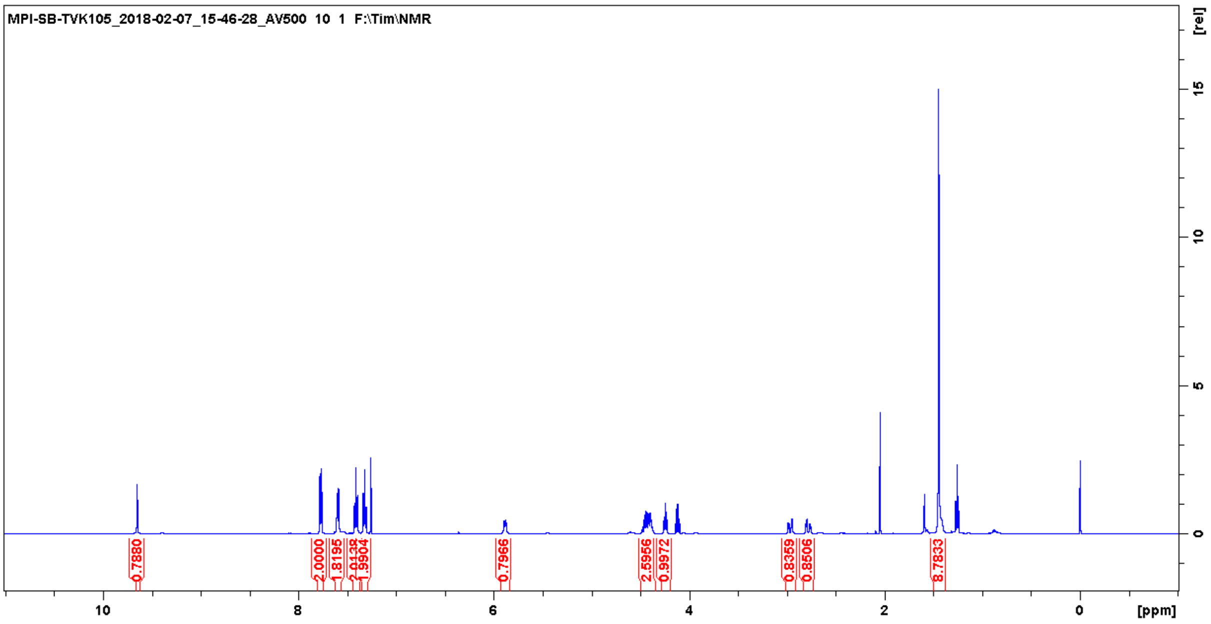

^{1}H NMR spectrum of compound **3**

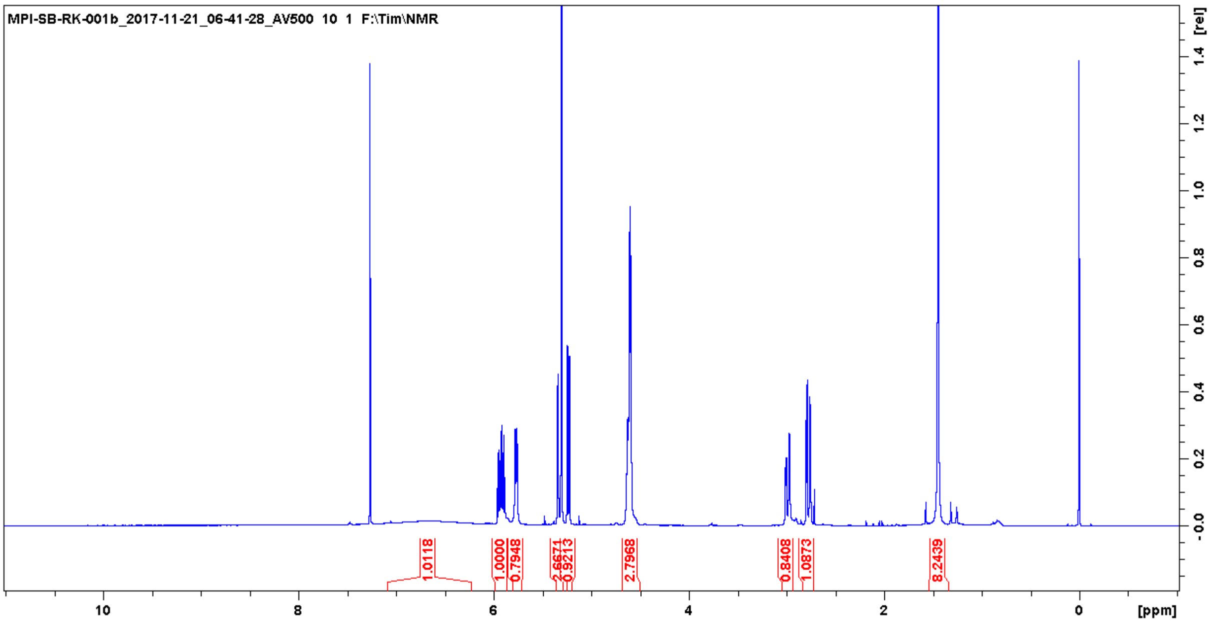

^{1}H NMR spectrum of compound **4**

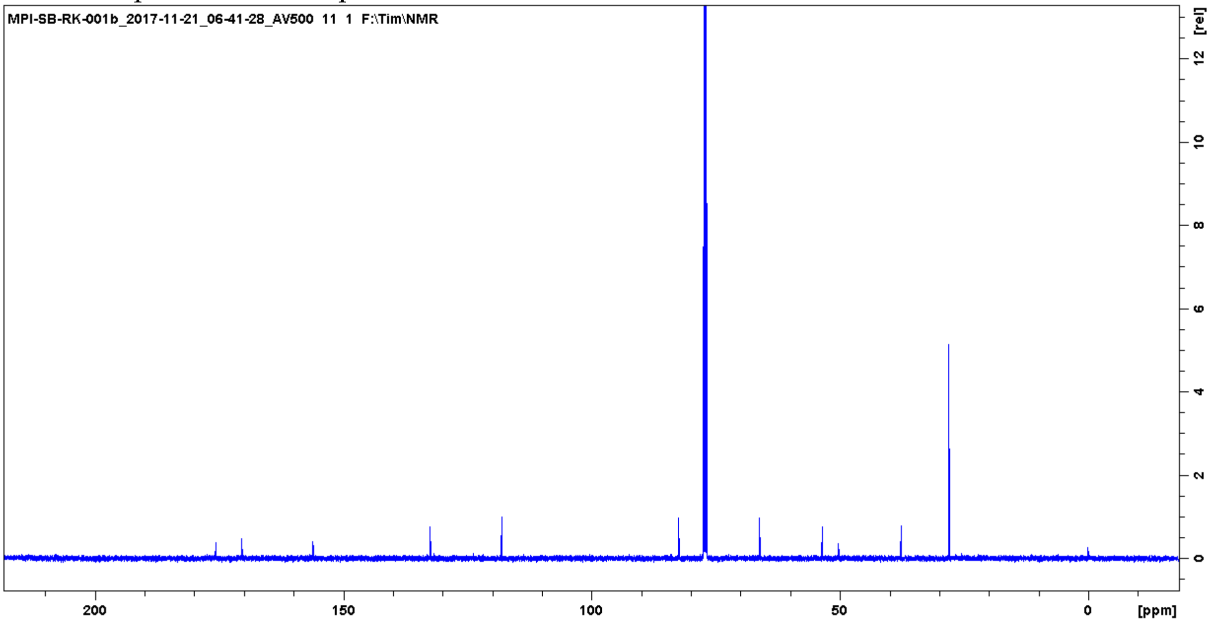

^{13}C NMR spectrum of compound **4**

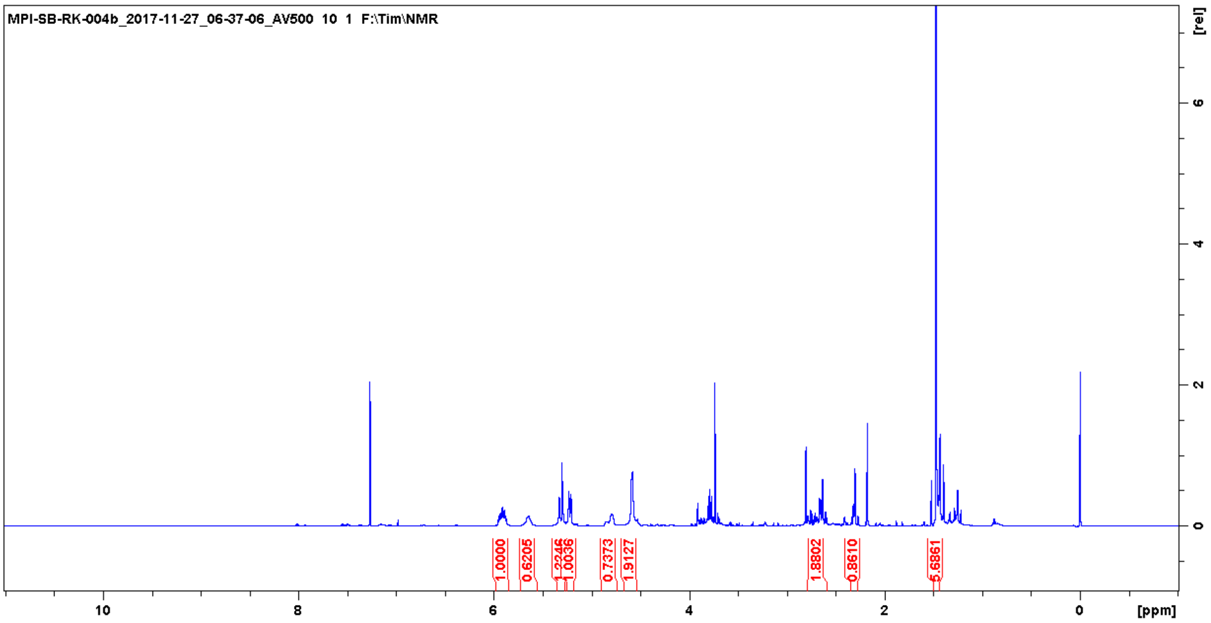

^{1}H NMR spectrum of compound **7**

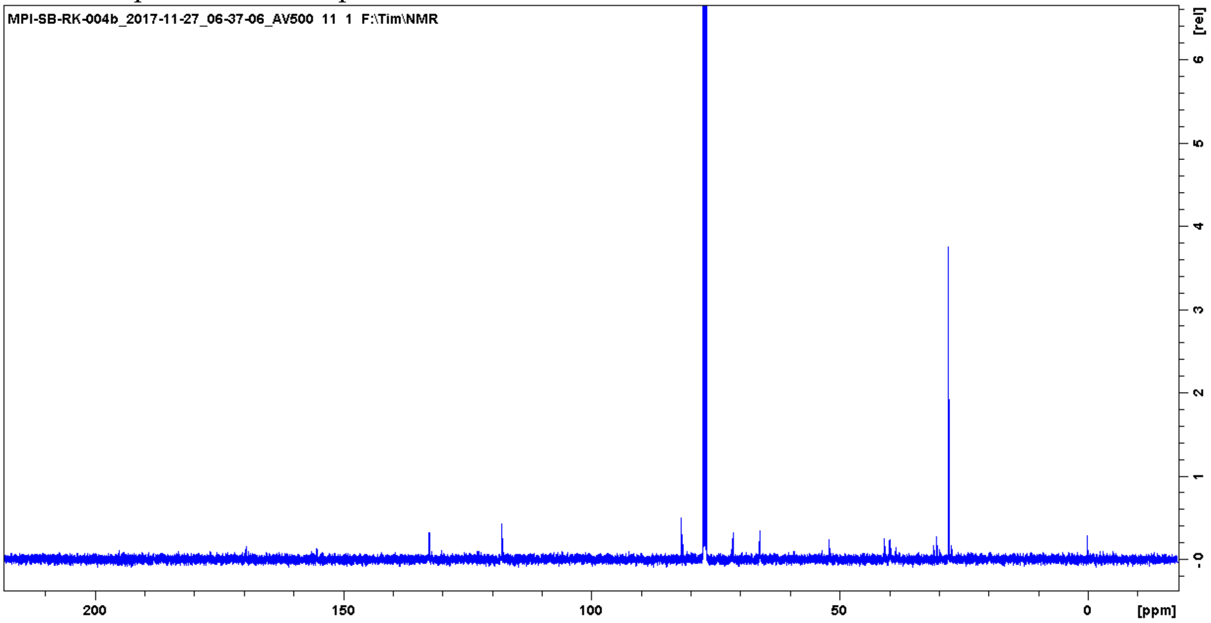

^{13}C NMR spectrum of compound **7**

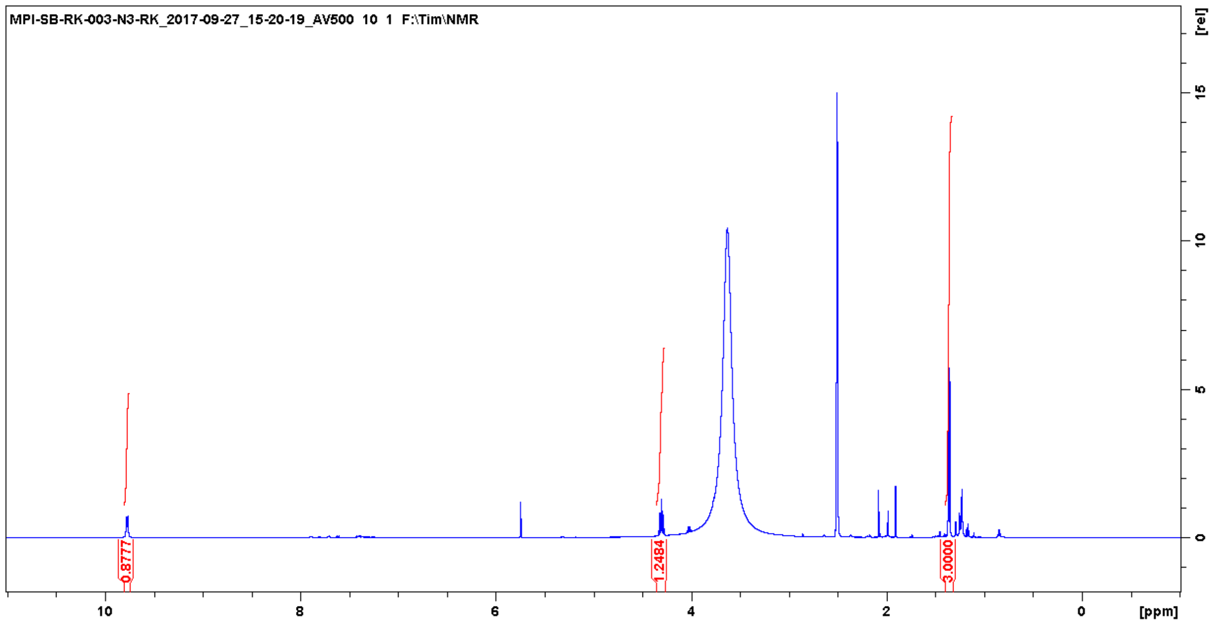

^{1}H NMR spectrum of compound **8**

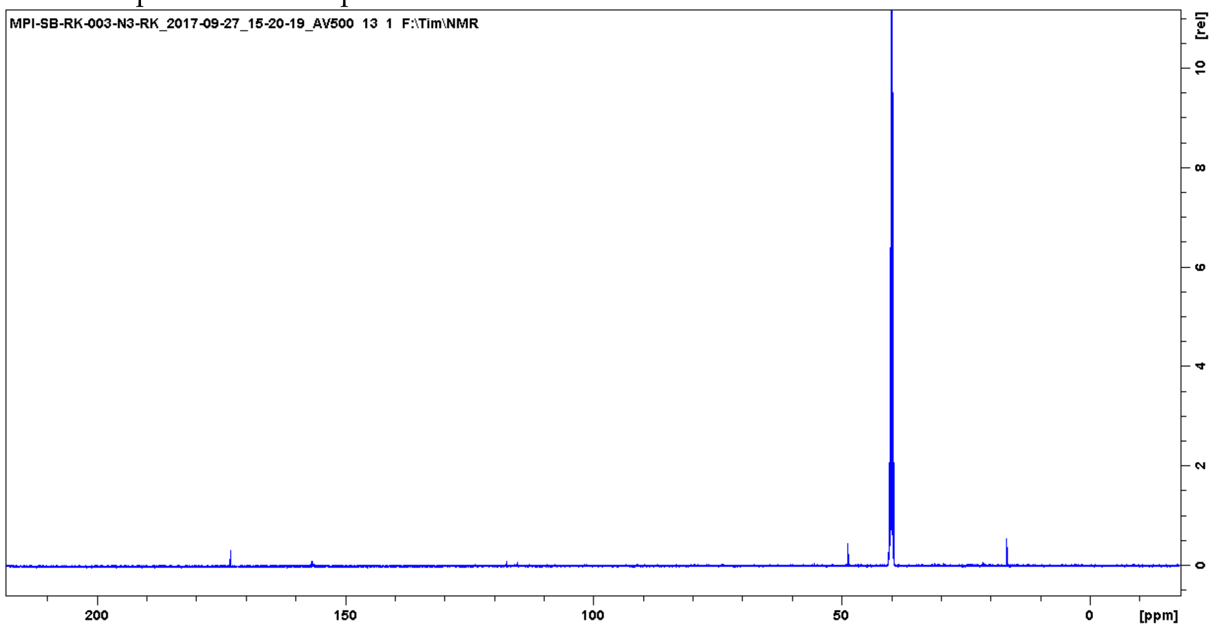

^{13}C NMR spectrum of compound **8**

Copies of LC-MS data

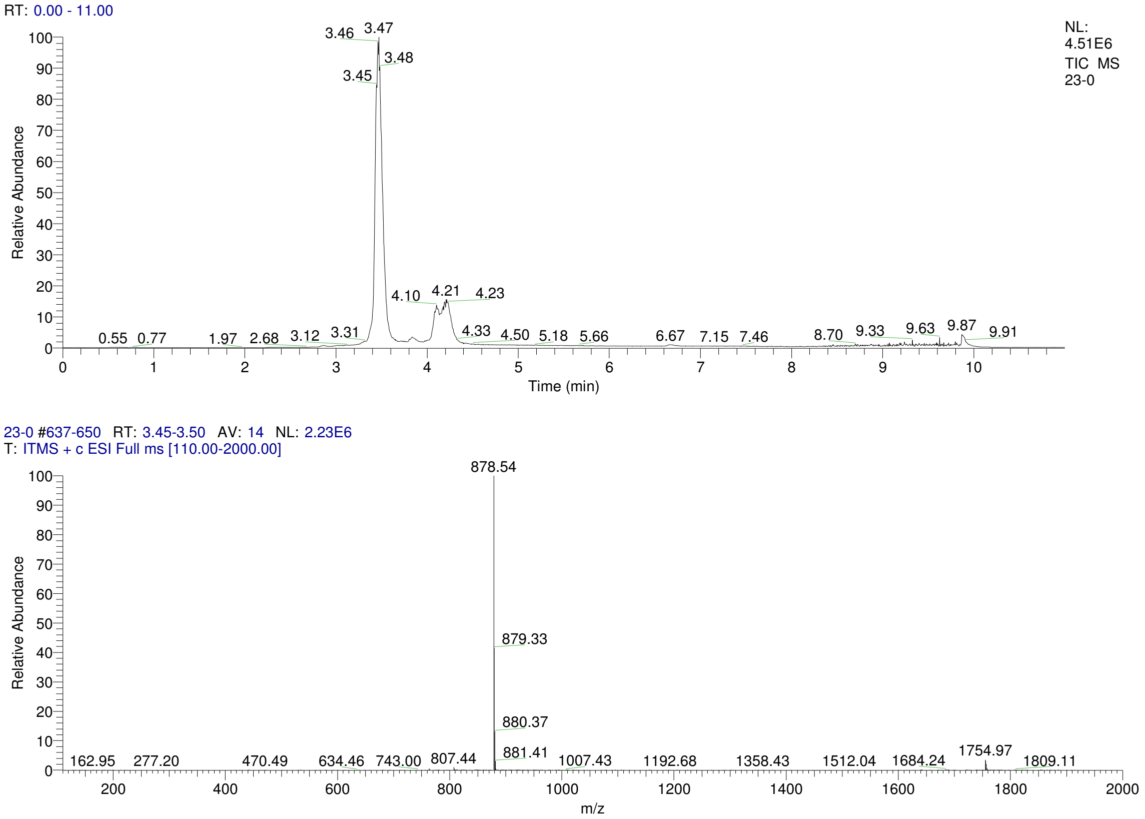

LC-MS data of probe **9a**

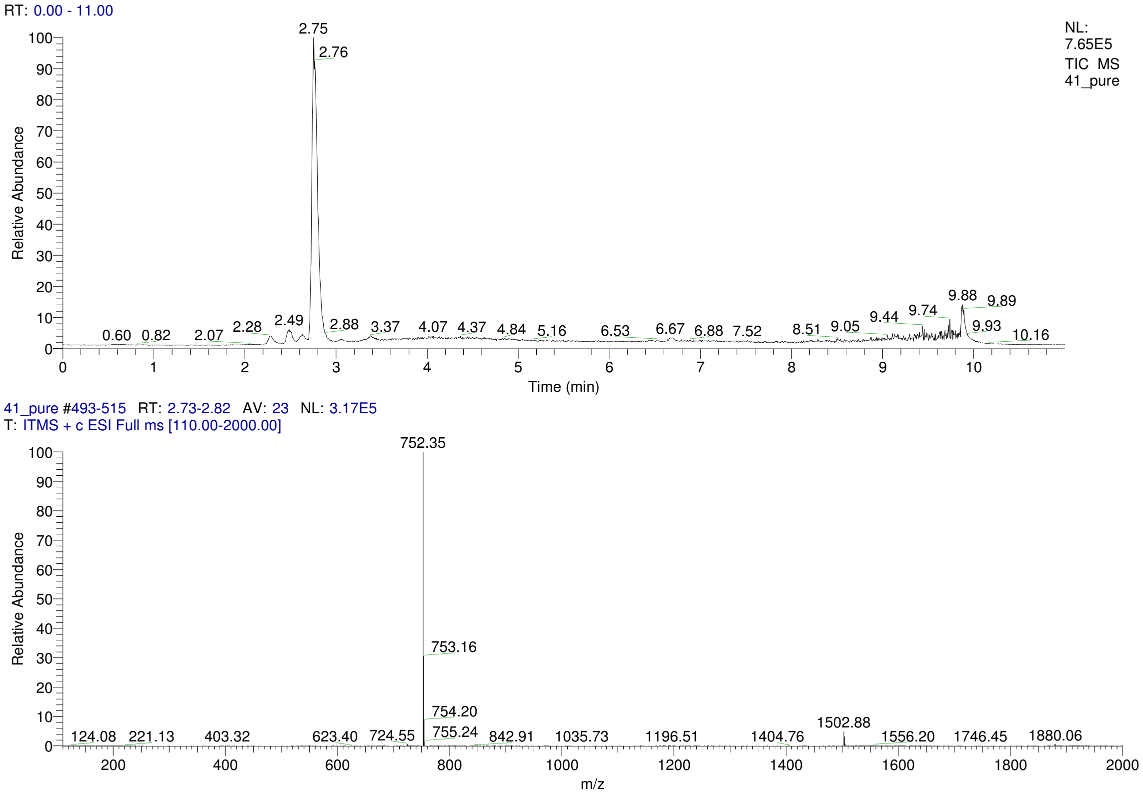

LC-MS data of probe **9b**

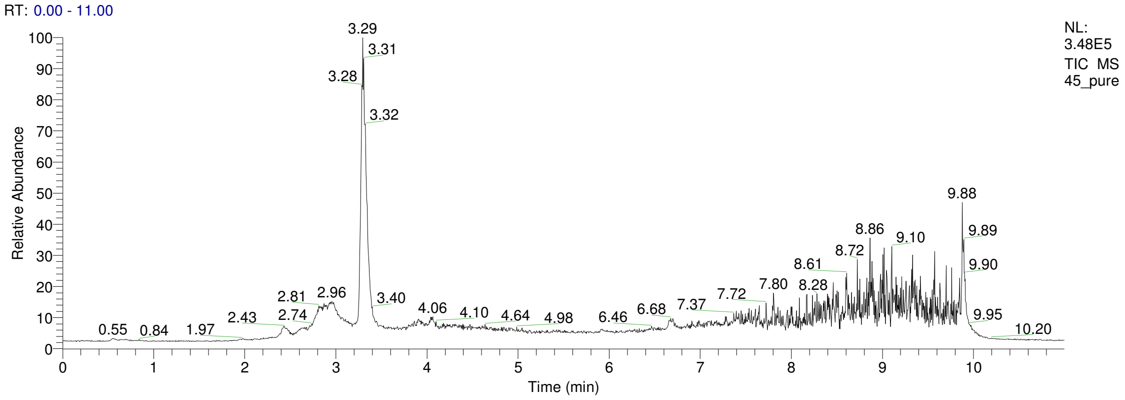

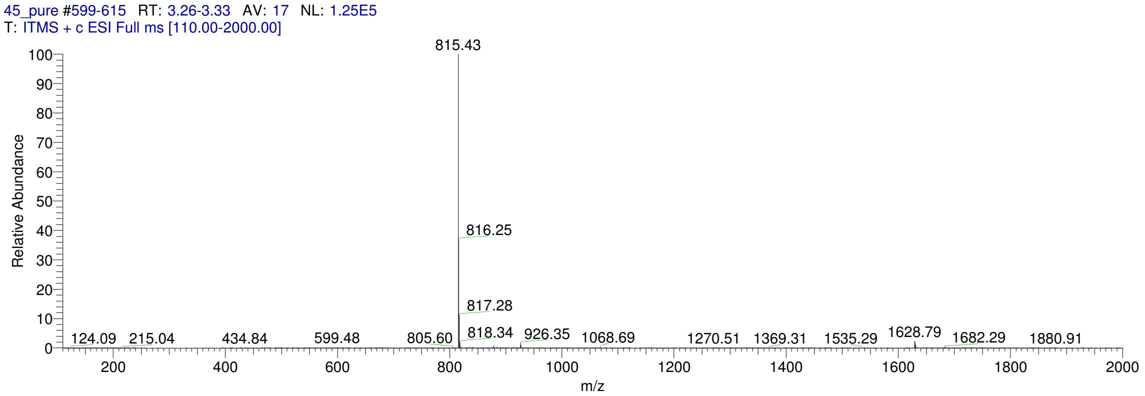

LC-MS data of probe **9c**

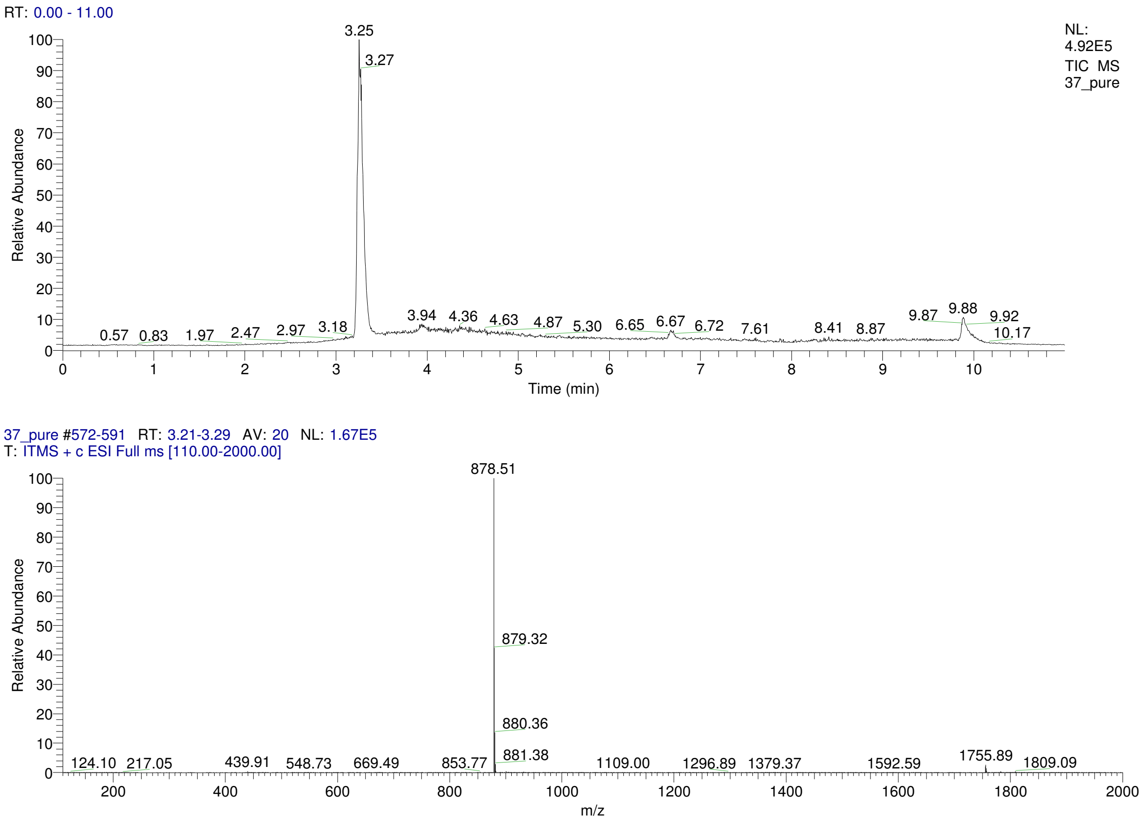

LC-MS data of probe **10a**

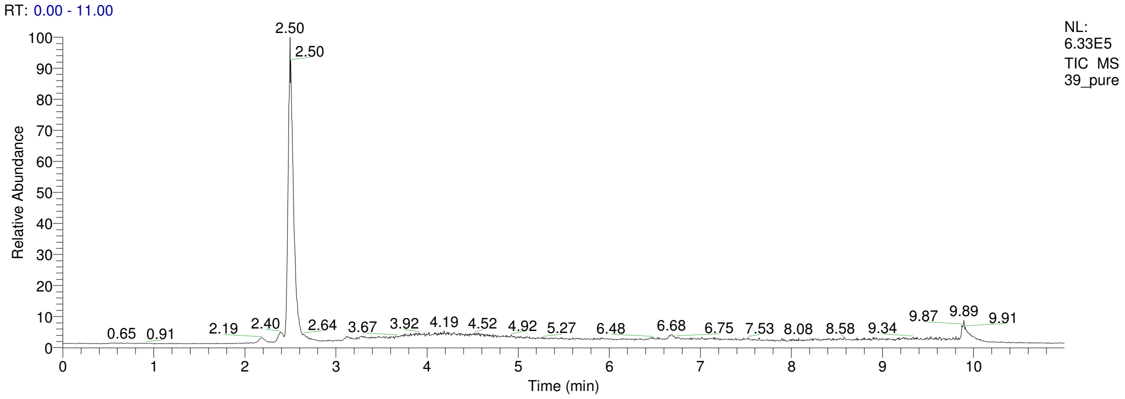

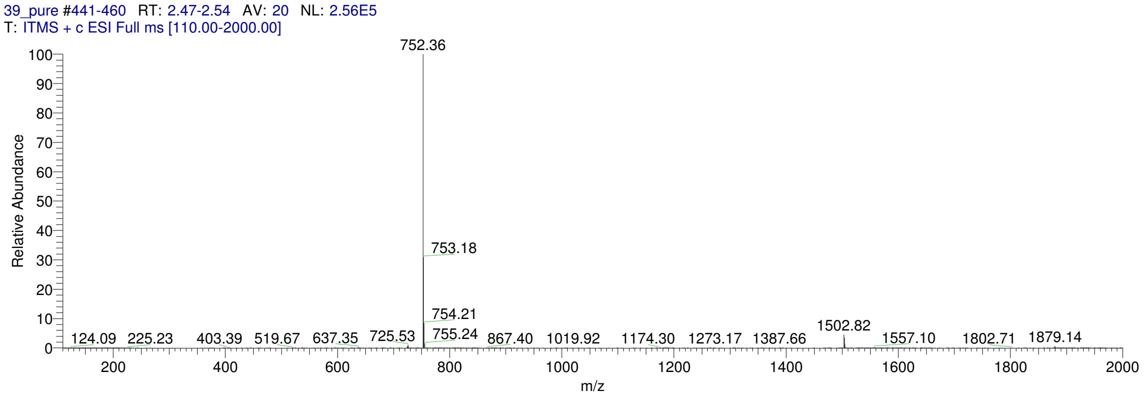

LC-MS data of probe **10b**

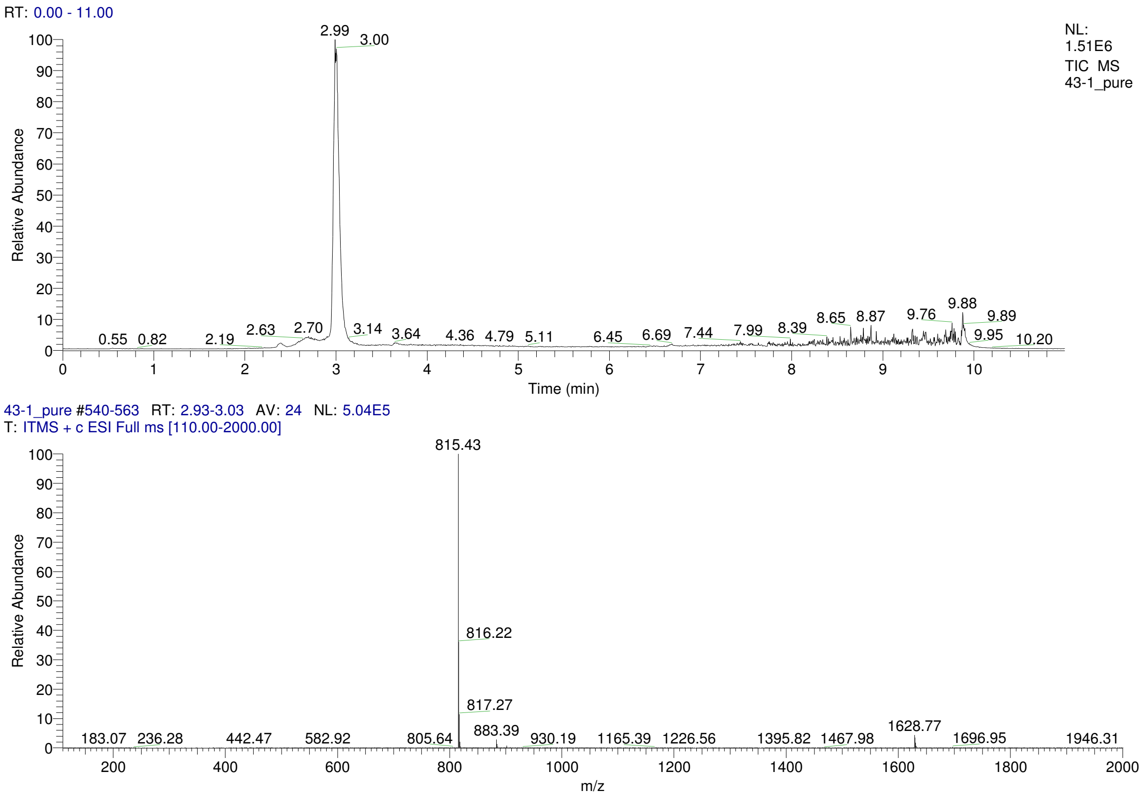

LC-MS data of probe **10c**

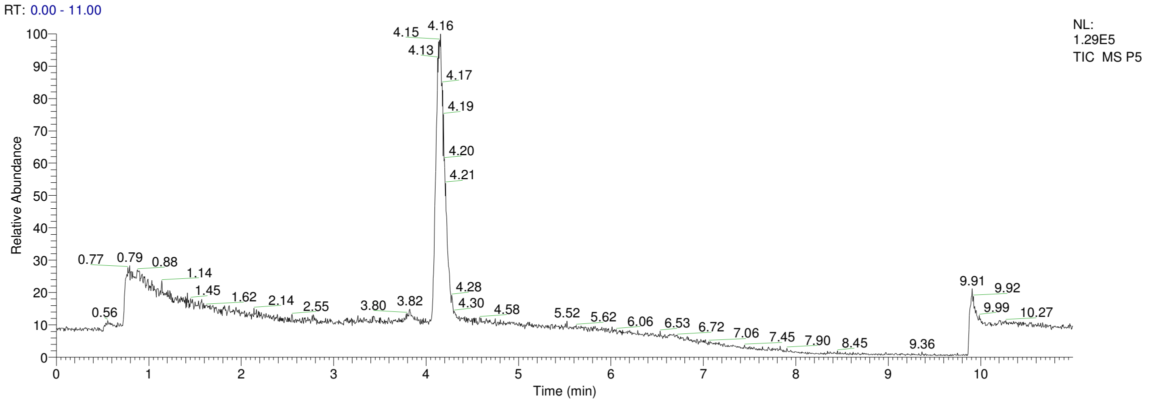

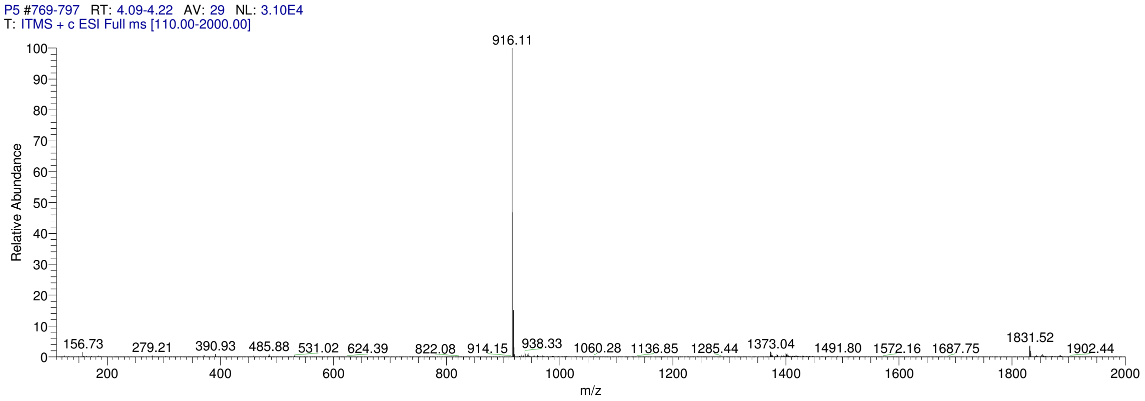

LC-MS data of probe **11a**

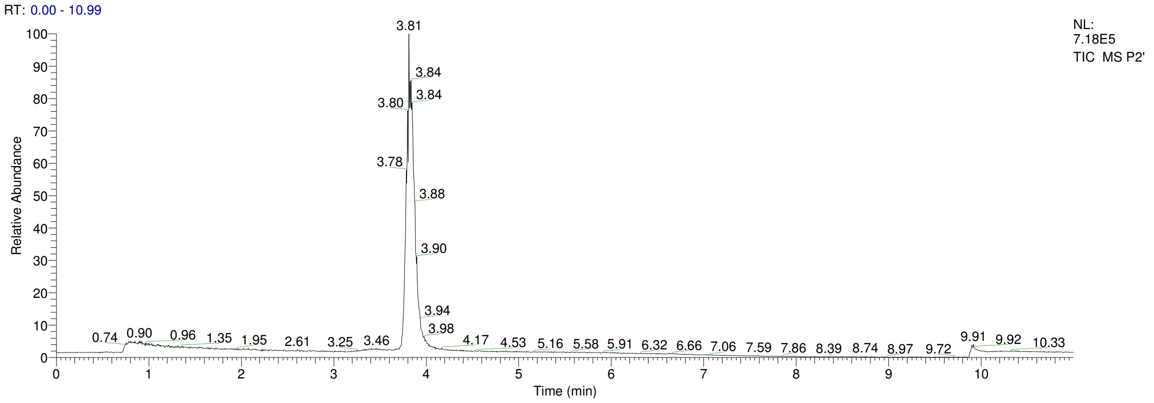

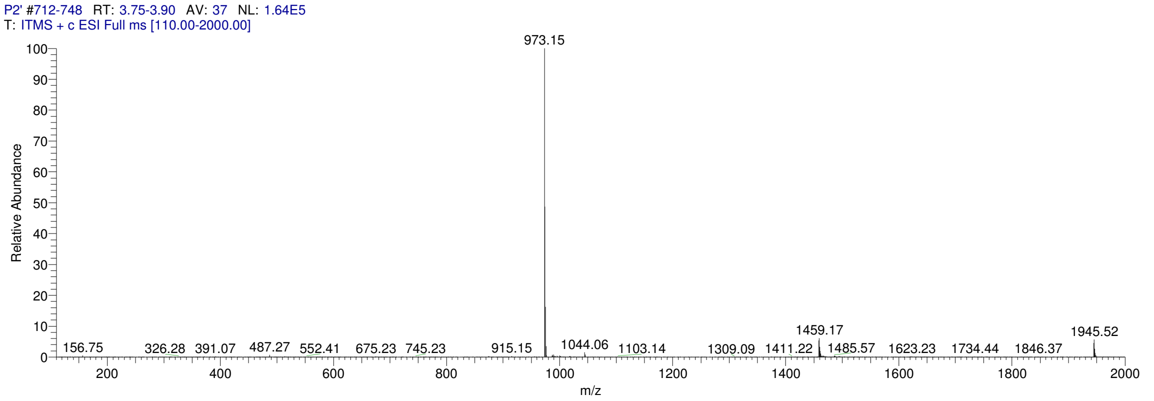

LC-MS data of probe **12a**
